# Neural Dynamics of Social Cognition: A Single‐Trial Computational Analysis of Learning Under Uncertainty

**DOI:** 10.1002/hbm.70433

**Published:** 2026-01-14

**Authors:** Colleen E. Charlton, Daniel J. Hauke, Vladimir Litvak, Michelle Wobmann, Renate de Bock, Christina Andreou, Stefan Borgwardt, Volker Roth, Andreea O. Diaconescu

**Affiliations:** ^1^ Krembil Centre for Neuroinformatics Centre for Addiction and Mental Health (CAMH) Toronto Ontario Canada; ^2^ Hawkes Institute, Department of Computer Science University College London London UK; ^3^ Department of Imaging Neuroscience University College London London UK; ^4^ Department of Psychiatry (UPK) University of Basel Basel Switzerland; ^5^ Department of Psychiatry and Psychotherapy, Translational Psychiatry University of Lübeck Lübeck Germany; ^6^ Department of Mathematics and Computer Science University of Basel Basel Switzerland; ^7^ Institute of Medical Sciences University of Toronto Toronto Ontario Canada; ^8^ Department of Psychiatry University of Toronto Toronto Ontario Canada; ^9^ Department of Psychology University of Toronto Toronto Ontario Canada

**Keywords:** EEG, functioning, hierarchical Gaussian filter, predictive coding, social learning, theory of mind

## Abstract

Understanding others' intentions amidst uncertainty is critical for effective social interactions, yet the neural mechanisms underlying this process are not fully understood. Here, we combined computational modeling and single‐trial EEG analysis to examine how the brain dynamically updates beliefs about others' intentions in volatile social contexts. A total of 43 healthy volunteers engaged in a deception‐free advice‐taking task, featuring alternating stable and volatile phases that systematically manipulated the reliability of an adviser's intentions. Using the hierarchical Gaussian filter (HGF), a Bayesian model of learning, we quantified trial‐by‐trial updates of participants' beliefs and their neural correlates. EEG amplitudes systematically varied according to task volatility, engaging neural regions associated with uncertainty processing such as the fusiform gyrus and posterior cingulate cortex. Sensor‐level EEG analyses confirmed a temporal sequence consistent with the hierarchical computations predicted by the HGF, whereby lower‐level prediction errors were processed earlier than higher‐order volatility‐related signals. Moreover, individual differences in these hierarchical neural processes correlated significantly with psychosocial functioning, suggesting that disruptions in Bayesian belief updating may underlie functional impairments in clinical populations. Collectively, our results reveal novel neural evidence for hierarchical Bayesian inference during social learning, highlighting its critical role in adaptive social behavior and potential relevance to mental health.

## Graphical abstract

We apply a hierarchical Bayesian model to single‐trial EEG data from an advice‐taking task, demonstrating that EEG activity closely mirrors the predicted sequence of prediction errors and precision signals. Additionally, source‐localised activity in the fusiform gyrus and posterior cingulate cortex covaried with participants' psychosocial functioning.

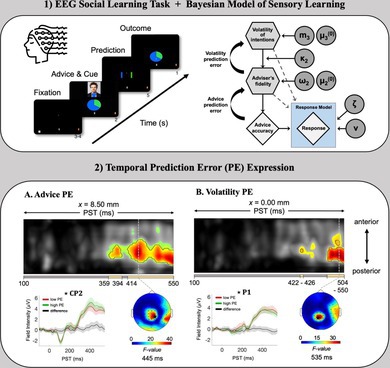

## 1 Introduction

Learning about others' intentions is a fundamental yet complex component of social interactions, involving the interpretation of ambiguous behaviors and actions. Social cues are inherently dynamic and context‐sensitive, posing significant challenges in social decision‐making processes such as whom to trust, how to communicate, and predicting others' reactions (Frith and Frith 2006; Malle and Holbrook 2012; FeldmanHall and Shenhav 2019). Effectively navigating this ambiguity is crucial for social learning, as misinterpretation may lead to misunderstandings or, in extreme cases, the development of false beliefs, such as paranoid delusions (Diaconescu et al. 2019; Frick et al. 2020).

A key dimension of social uncertainty is the perceived volatility of others' intentions, the rate at which these intentions change over time. Here, volatility refers to changes in the adviser's underlying strategy over time (e.g., shifting from mostly honest to mostly misleading recommendations) (Diaconescu et al. 2014; Diaconescu, Litvak, et al. 2017), whereas stochasticity denotes the within‐policy noise in outcomes or advice (e.g., being 60% vs. 80% honest at a given time). Based on the original paradigm (see Diaconescu et al. 2014), our paradigm manipulates volatility via incentive‐driven policy shifts while aiming to keep stochasticity approximately constant (privileged‐information accuracy ≈80%). However, because advice was replayed from real human advisers recorded during a prior face‐to‐face interaction (Diaconescu et al. 2014), volatility and stochasticity could not be orthogonally manipulated; small stochastic fluctuations necessarily arise from human decision variability even within a given policy (see Section 4.4). High volatility introduces additional complexity, as individuals must distinguish between stable behavioral patterns and rapidly shifting motives (Shin et al. 2019). This dynamic nature of volatility often necessitates hierarchical inference to adaptively update beliefs about others' intentions in response to fluctuating social environments (Diaconescu et al. 2014).

The dynamic interplay between volatility and hierarchical belief updating underscores the importance of adaptive learning in social cognition. Bayesian theories provide a compelling framework for understanding how the brain processes uncertainty and revises beliefs about others' intentions (Diaconescu et al. 2014; Barnby et al. 2023). According to hierarchical Bayesian theory, the brain continuously generates and updates beliefs about the environment based on prediction errors (PEs)—the difference between expected and observed outcomes (Friston 2005; Friston and Stephan 2007; Aitchison and Lengyel 2017). These beliefs are organized hierarchically, ranging from simple sensory interpretations to more complex, higher‐order environmental statistics (Kiebel et al. 2008). The precision of these PEs reflects their reliability and is used to dynamically adjust the learning rate, determining how much beliefs are updated in response to new evidence (Wacongne et al. 2011; Mathys et al. 2011, 2014; Aitchison and Lengyel 2017). Thus, in generic hierarchical Bayesian frameworks—such as the hierarchical Gaussian filter (HGF)—a ratio of these precisions controls the influence of PEs on belief updating, prioritizing lower‐level (sensory) inputs when they are precise relative to higher‐level predictions (Mathys et al. 2011, 2014).

The challenge of accurately inferring others' intentions is particularly pronounced when distinguishing honest mistakes from deliberate misinformation. In our paradigm, volatility refers specifically to changes in the adviser's underlying policy (e.g., shifting from being mostly honest to mostly misleading). This process is further complicated by the uncertainty, including volatility, inherent to social interactions. Previous research has linked impairments in estimating environmental volatility to conditions such as autism (Lawson et al. 2017), schizophrenia (Deserno et al. 2020; Fromm et al. 2023), and paranoia specifically (Diaconescu et al. 2019; Reed et al. 2020; Wellstein et al. 2020; Suthaharan et al. 2021; Hauke et al. 2024; Karvelis et al. 2024). Despite this progress, the temporal dynamics of hierarchical belief updating during social interactions remain largely unexplored, particularly at the neural level (FeldmanHall and Nassar 2021).

To address this gap, we employed the HGF model (Mathys et al. 2011, 2014)—a hierarchical Bayesian model of learning—to analyze single‐trial electroencephalography (EEG) data from healthy volunteers engaged in an established social learning task. This computational approach allowed us to capture trial‐wise estimates of different forms of uncertainty and PEs, providing a detailed account of how the brain dynamically adapts to changing social cues over time. We hypothesised that the volatility of the adviser's strategy would significantly impact the amplitude of the feedback‐related negativity (FRN) component, such that unexpected outcomes during stable phases—where predictions about the adviser's behavior are stronger—would elicit larger (more negative) FRN amplitudes compared to volatile phases, where expectations are inherently weaker due to frequent changes (Sambrook and Goslin 2015; Walsh and Anderson 2012). Additionally, we hypothesised that social belief formation can be modelled as Bayesian belief updating with the HGF. This model predicts a specific computational sequence, whereby certain quantities are computed only after others have been evaluated. Consequently, we anticipated that EEG data would reflect this computational hierarchy, with neural response timing aligning with the sequential computational steps predicted by the HGF (Diaconescu, Mathys, et al. 2017).

In our social learning task, participants engaged in a binary lottery game, integrating non‐social information (a visual display of winning probabilities) with advice from an adviser possessing additional information but varying incentives to either assist or mislead the participant. This design requires hierarchical belief updating about advice accuracy, adviser fidelity, and specifically the volatility of intentions.

Our study extends previous social learning research based on behavioral outcomes (Diaconescu et al. 2014; Hauke et al. 2024) and fMRI data (Diaconescu, Mathys, et al. 2017) by examining the real‐time adaptive processes during active social interactions using EEG. We propose that the brain processes social learning hierarchically and temporally, consistent with HGF predictions. According to this model, during each trial, the brain computes six key quantities, PEs and precisions, in a specific temporal sequence: first the lower‐level computations (advice PE, advice precision, cue PE, and outcome PE, whose exact order may vary), followed by volatility PE and volatility precision (Diaconescu, Mathys, et al. 2017).

Finally, we conducted exploratory analyses to investigate the relationship between these computational processes and psychosocial functioning. We hypothesized that individuals demonstrating greater learning from advice would exhibit better psychosocial functioning, suggesting a mechanistic link between efficient social learning processes and mental health outcomes.

## 2 Materials and Methods

### 2.1 Participants

A total of 43 healthy controls (HC) aged 15 and above were recruited via online and public advertisements in Basel, Switzerland, in accordance with Swiss guidelines for minimal‐risk behavioral research in competent adolescents. Participants were initially recruited as part of a larger study probing delusion formation in early psychosis (Hauke et al. 2024). Here, we focus on the results from HCs due to insufficient EEG data from patients. Criteria for inclusion and exclusion are detailed in Appendix S1. All participants provided written informed consent and written consent from at least one legal guardian whenever the participant was < 18 years old. The study was conducted in accordance with the Declaration of Helsinki and approved by the local ethics committee of Northwestern Switzerland (Ethikkommission Nordwest‐ und Zentralschweiz, no. 2017‐01149).

### 2.2 Demographic Variables and Functioning Assessment

Within a five‐day window of EEG data collection, interviews were conducted to gather demographic information and assess functioning (Table 1). Functioning was evaluated using the Global Functioning: Social (GF: Social) and the Global Functioning: Role (GF: Role) scales (Cornblatt et al. 2007). GF: Social evaluates the quality of social interactions and participation in interpersonal activities, while GF: Role assesses an individual's effectiveness in work, educational, or home settings, depending on the age of the individual. Each scale was scored by trained raters, with higher scores denoting better functioning. Median scores for both GF: Social and GF: Role were 9, ranging from 6 to 10.

**TABLE 1:** Participant characteristics. Demographic characteristics and psychosocial functioning of the study sample. Mean values, and in parentheses standard deviation, are provided for continuous demographic variables, while psychosocial functioning is represented by median, 25th, and 75th percentiles.

	Healthy controls
Age (mean [SD])	22.9 [6.8]
Years of education (mean [SD])	13.2 [3.3]
Working memory^a^ (mean [SD])	16.4 [3.6]
Sex (f/m)	20/23
Handedness (l/*r*)	5/38
Cannabis (y/n)	27/16
Global functioning: social and role scales (Cornblatt et al. 2007)	
GF: Social (median [25th, 75th])	9 [8, 9]
GF: Role (median [25th, 75th])	9 [8, 9]

^a^ Assessed with the digit span backwards task from the Wechsler Adult Intelligence Scale‐Revised (Wechsler 1981).

### 2.3 Social Learning Task Design

**FIGURE 1:**
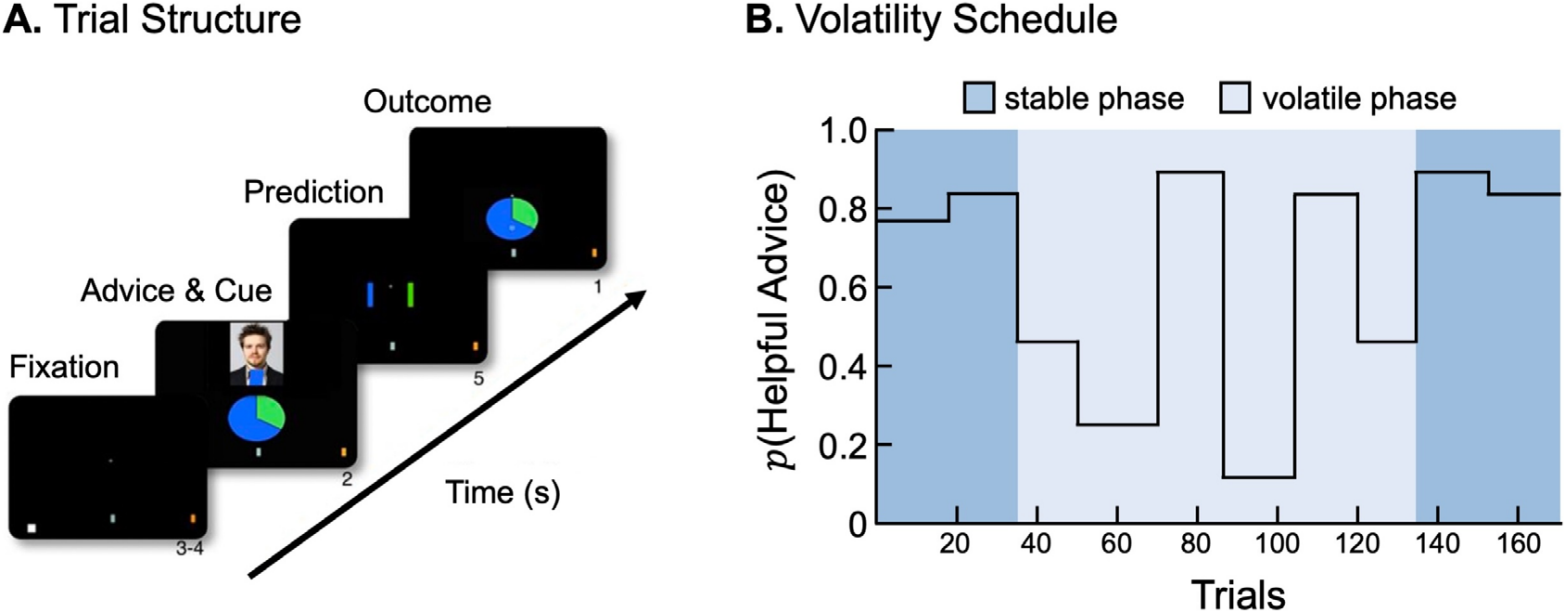
Social learning paradigm. (A) Participants (players) took part in an advice‐taking game for monetary rewards, where they predicted the outcome of a binary lottery. The adviser, whose recommendations were pre‐recorded and presented via video, had access to additional but incomplete information about the outcome. Depending on the adviser's changing incentives throughout the game, they could offer either helpful or misleading advice. Participants were truthfully informed that the adviser's intentions might shift over the course of the experiment, either due to errors or a different agenda, and that the adviser's strategy could alternate between assisting and misleading the player. (B) The volatility schedule of the adviser's intentions. Initially, advice was predominantly stable and helpful. In the volatile phase, the adviser's intentions changed more frequently, altering the reliability of the advice.

Participants engaged in an ecologically valid, deception‐free social learning task (Diaconescu et al. 2014; Diaconescu, Litvak, et al. 2017), in which they predicted the outcomes of a binary lottery (similar to Behrens et al. 2008). They had to integrate information from a visual cue, displaying accurate winning probabilities, and advice from an adviser whose intentions varied throughout the experiment (Figure 1A). The task included stable phases, where the adviser consistently provided helpful advice, and a volatile phase, marked by rapid shifts in the adviser's intentions (Figure 1B).

The task comprised 170 trials, each featuring distinct visual cues presented as pie charts representing probabilistic outcomes. The adviser's recommendations were delivered through pre‐recorded video clips derived from authentic human‐to‐human interactions, where the advisers genuinely attempted to either help or mislead the participants based on their incentives. The adviser's recommendations were replayed from a real‐time, face‐to‐face dyadic version of the task (Diaconescu et al. 2014). Policy volatility was induced by block‐level incentive changes (to shift from cooperative to competitive strategies), which alter the adviser's intention to help versus mislead. Stochasticity was constrained by the adviser's privileged‐information accuracy (80%) and by the non‐social cue (pie‐chart base rate). Because the replayed sequences reflect human advisers' actual trial‐by‐trial decisions, we could not independently vary within‐policy consistency and the timing/magnitude of policy shifts; hence, volatility and stochasticity are not orthogonal by design. Analyses therefore focus on volatility‐related learning while acknowledging residual within‐policy noise. Participants were truthfully informed that the adviser possessed privileged but incomplete information about the outcomes, and that inaccurate advice could result either from unintentional errors or intentional changes in the adviser's motives, further enhancing the ecological validity of the task. It was clear to participants that the advice was pre‐recorded rather than a live interaction.

Participants were asked to predict the outcome of the lottery on each trial based on this information. Feedback was provided on each trial, and participants' prediction performance determined their final payment. All participants earned a base rate of 25 CHF per hour, with additional bonuses of 10 CHF for meeting a predefined “silver” accuracy target (which was achieved on average) and 20 CHF for meeting a predefined “gold” accuracy target.

The task was implemented in MATLAB R2018a (https://www.mathworks.com) using Cogent 2000 v1.32 for stimulus delivery and response logging. Auditory and visual stimuli were rendered with PsychToolbox‐3 (v3.0.14).

### 2.4 Computational Modeling Framework

**FIGURE 2:**
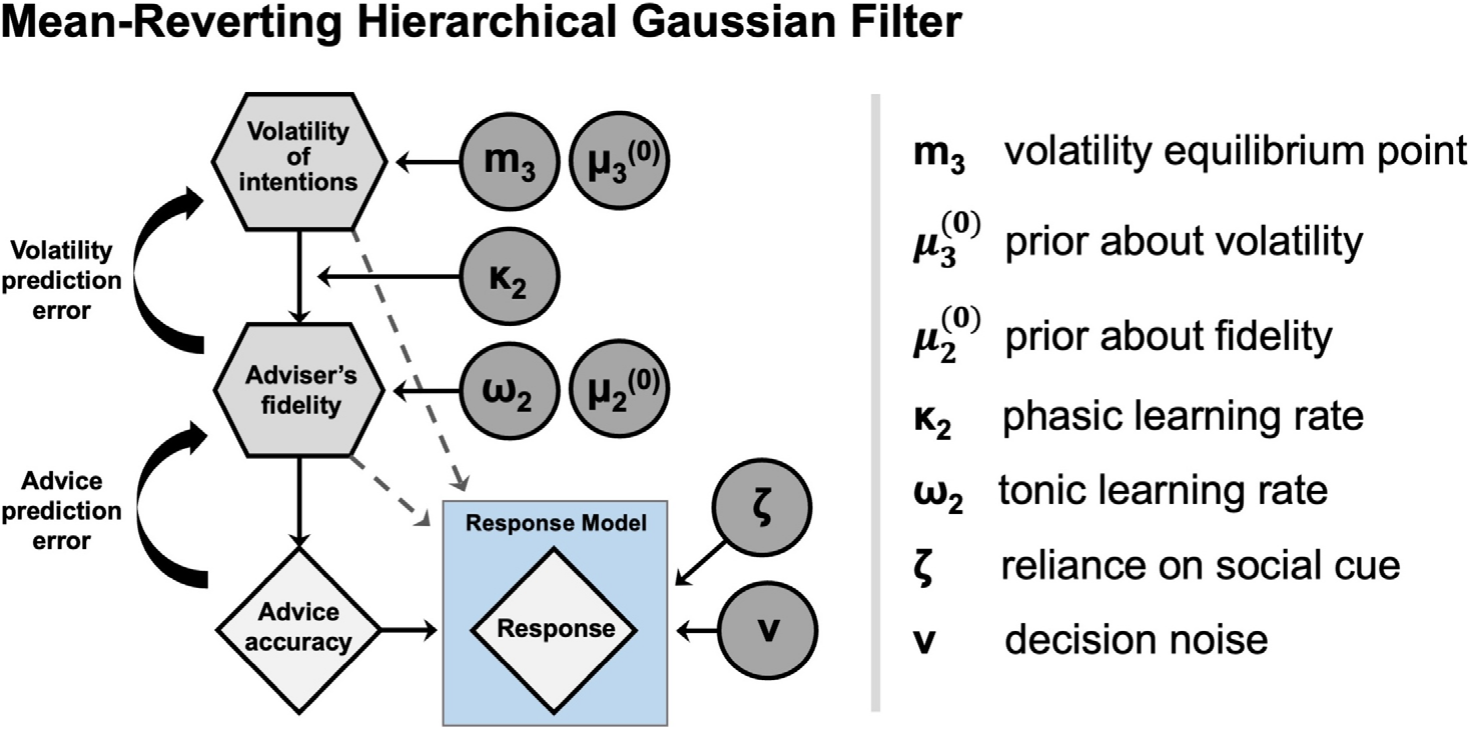
Mean‐reverting hierarchical Gaussian filter (HGF). This diagram illustrates a three‐level HGF with a drift component at the third level, designed to capture learning about the volatility of the adviser's intentions. Parameters emphasized in the figure represent the free parameters within the model.

Consistent with prior research on learning in volatile environments (Diaconescu et al. 2014; Deserno et al. 2020; Hauke et al. 2024; Karvelis et al. 2024), we model participants' behavior using the hierarchical Gaussian filter (HGF) model (Figure 2). We used the HGF to capture hierarchical belief updating about advice reliability and its volatility at a higher level, consistent with our policy‐change manipulation and prior applications of this paradigm (Diaconescu et al. 2014; Diaconescu, Litvak, et al. 2017). Importantly, in the original face‐to‐face task (Diaconescu et al. 2014), where the adviser's intentionality (intentional vs. blindfolded card‐selection) was explicitly manipulated across conditions, the HGF model family uniquely captured, within that study, intentional advice‐giving via hierarchical inference. By contrast, a standard Kalman filter assumes constant process noise and thus cannot represent changes in volatility without additional mechanisms (Piray and Daw 2020). Our choice of HGF therefore directly targets the construct manipulated by the task (policy volatility) and aligns with evidence that hierarchical inference is necessary to capture intentional advice‐giving in this paradigm.

Based on previous research (Hauke et al. 2024), we considered two perceptual models, the binary HGF—a hierarchical Bayesian model for learning under uncertainty (Mathys et al. 2011, 2014)—and a mean‐reverting HGF. This second model includes a drift in volatility estimates towards a subject‐specific equilibrium, thereby capturing three possible trajectories: an *upward drift* (perceiving the environment as increasingly volatile over time), a *downward drift* (perceiving it as increasingly stable) or a *reversion to the prior* (i.e., “forgetting” previously observed inputs).

Conceptually, these models differ in how they represent volatility and its impact on learning. The binary HGF assumes that participants learn about advice fidelity and volatility solely based on trial‐by‐trial updates from empirical observations. In contrast, the mean‐reverting HGF incorporates an additional drift mechanism, where volatility estimates gradually revert towards a subject‐specific equilibrium point, regardless of empirical observations. This equilibrium represents a baseline expectation about environmental volatility. We used the HGF framework because our task was designed to manipulate volatility, which requires a hierarchical model that can track both advice reliability and changes in underlying policy. Standard models such as the Kalman filter assume a stationary environment and therefore cannot capture these higher‐level belief updates.

This comparison allows us to test whether human learners rely purely on data‐driven updates or if they also incorporate a prior expectation about volatility over time (Shin et al. 2019). The mean‐reverting process reflects the hypothesis that individuals may maintain a default belief about the volatility of others' intentions, which could modulate their sensitivity to observed changes. By comparing these models, we can assess whether participants adapt their learning rates dynamically based on empirical data alone or whether this adaptation is constrained by a latent stability bias. Notably, this model was previously favored in at‐risk for psychosis individuals and patients with known social learning deficits (Cole et al. 2020; Hauke et al. 2024).

#### 2.4.1 Perceptual Model: Hierarchical Gaussian Filter

The standard 3‐level HGF (Mathys et al. 2011, 2014) assumes that hidden states at each level (denoted as $x_{1}^{\left(t\right)}$, $x_{2}^{\left(t\right)}$, …, $x_{n}^{\left(t\right)}$, where *t* is the trial index and *n* the number of hierarchy levels) evolve over time as hierarchically coupled Gaussian random walks. The step size (variance) at each level is controlled by the level above.

In the context of our task, participants infer the congruence between the advice and the observed outcome $u^{\left(t\right)}$. The lowest state, $x_{1}$, described by a Bernoulli distribution, represents the *advice accuracy* on each trial, being either accurate ($u^{\left(t\right)}=1$) or inaccurate ($u^{\left(t\right)}=0$). $x_{1}$ is probabilistically linked to the state at the second level, $x_{2}$, through a logistic sigmoid transformation:

(1) $$p\left(x_{1}^{\left(t\right)}|x_{2}^{\left(t\right)}\right)=s{\left(x_{2}^{\left(t\right)}\right)}^{x_{1}^{\left(t\right)}}{\left(1-s\left(x_{2}^{\left(t\right)}\right)\right)}^{1-x_{1}^{\left(t\right)}}∼\text{Bernoulli}\left(x_{1}^{\left(t\right)};s\left(x_{2}^{\left(t\right)}\right)\right)$$

where

(2) $$s\left(z\right)≝\frac{1}{1+\exp\left(-x\right)}$$

State $x_{2}$ reflects the *adviser's fidelity* or the tendency towards providing helpful advice, whereas the highest state $x_{3}$ reflects how quickly the intentions of the adviser ($x_{2}$) are changing over time, i.e., *volatility of the adviser's intentions*. Both states are specified by normal distributions (Equations (3) and (4), respectively).

(3) $$p\left(x_{2}^{\left(t\right)}|x_{2}^{\left(t-1\right)},x_{3}^{\left(t\right)},\kappa_{2},\omega_{2}\right)∼N\left(x_{2}^{\left(t\right)};x_{2}^{\left(t-1\right)},\exp\left(\kappa_{2}x_{3}^{\left(t\right)}+\omega_{2}\right)\right)$$

(4) $$p\left(x_{3}^{\left(t\right)}|x_{3}^{\left(t-1\right)},\vartheta \right)∼N\left(x_{3}^{\left(t\right)};x_{3}^{\left(t-1\right)},\vartheta \right)$$

The dynamics of these states are shaped by three subject‐specific parameters. Parameter $\kappa_{2}$, determines the coupling between the second level ($x_{2}$) and third level ($x_{3}$), reflecting how an individual uses their estimation of the adviser's changing intentions to evaluate current advice. Evolution rate parameter $\omega_{2}$ represents the baseline learning rate at the second level, independent of the phasic volatility at $x_{3}$. This parameter captures to what extent a participant updates beliefs about the adviser's fidelity, unaffected by the perceived volatility of the adviser's intentions. The meta‐volatility parameter ϑ is the variance of $x_{3}$. In line with previous studies, parameter ϑ was fixed to 0.5 to reduce the number of free parameters (Hauke et al. 2024; Karvelis et al. 2024). These parameters define each participant's learning style and the evolution of their beliefs.

#### 2.4.2 Perceptual Model: Mean‐Reverting Hierarchical Gaussian Filter

Following previous work (Cole et al. 2020; Hauke et al. 2024), we also employed the mean‐reverting HGF, which incorporates a drift parameter that encourages a return to equilibrium beliefs about environmental volatility, analogous to an Ornstein–Uhlenbeck process in discrete time (Uhlenbeck and Ornstein 1930). This model assumes that learning about an adviser's intentions is influenced not only by hierarchical PE updates but also by a drift in perceived volatility. Specifically, the drift parameter allows for three possible trajectories: shifts towards perceiving the environment as increasingly volatile over time, shifts towards perceiving the environment as increasingly stable over time, and a reversion to prior beliefs (i.e., “forgetting”) about environmental volatility if the equilibrium point $m_{3}$ is equal to the prior mean of volatility. In this mean‐reverting HGF, the third level can again be described by a normal distribution:

(5) $$p\left(x^{\left(t\right)}|x_{3}^{\left(t-1\right)},\vartheta ,\phi_{3},m_{3}\right)∼N\left(x_{3}^{\left(t\right)};x_{3}^{\left(t-1\right)}+\phi_{3}\left(m_{3}-x_{3}^{\left(t-1\right)}\right),\vartheta \right),$$

where $\phi_{3}$ denotes the drift rate and $m_{3}$ the equilibrium point that the state moves towards over time. Parameter $\phi_{3}$ was fixed to 0.1, while the equilibrium point $m_{3}$ was estimated as a subject‐specific, free parameter.

#### 2.4.3 Model Inversion: The Update Equations

The HGF uses a variational approximation for trial‐by‐trial updates of beliefs about states (Mathys et al. 2011, 2014). This process involves updating inferences after each observation, where the belief at each hierarchical level is adjusted based on the precision from the level below and the PE. Here, “belief” signifies a posterior probability distribution characterized by its sufficient statistics. Specifically, the belief update equations are proportional to precision‐weighted PEs (pwPEs):

(6) $$\Delta \mu_{i}^{\left(t\right)}\propto \frac{{\hat{\pi }}_{i-1}^{\left(t\right)}}{\pi_{i}^{\left(t\right)}}\delta_{i-1}^{\left(t\right)}$$

where the belief or expectation at trial *t* and level *i* of the hierarchy, denoted as $\mu_{i}^{\left(t\right)}$, is scaled by the precision ratio. This ratio consists of the precision estimate from the level below (${\hat{\pi }}_{i-1}^{\left(t\right)}$), where the hat symbol indicates that this value has not yet been updated based on new input and corresponds to the predicted precision before observing new input. This is then divided by the updated precision at the current level ($\pi_{i}^{\left(t\right)}$), multiplied by the PE from the level below ($\delta_{i-1}^{\left(t\right)}$). This approach is structurally similar to reinforcement learning models, but also introduces a dynamic learning rate governed by the higher level in the hierarchy.

At trial *t*, the outcome *u*, signifying accurate ($u^{\left(t\right)}=1$) or inaccurate ($u^{\left(t\right)}=0$) advice, initiates a hierarchical sequence of belief revisions as per the update equations. At the most basic level, observation $u^{\left(t\right)}$, posterior belief $\mu_{1}^{\left(t\right)}$, and state $x_{1}^{\left(t\right)}$ are equivalent due to unambiguous advice accuracy perceived by the participant:

(7) $$\mu_{1}^{\left(t\right)}=u^{\left(t\right)}=x_{1}^{\left(t\right)}$$

Nevertheless, the outcome $u^{\left(t\right)}$ generates an advice PE $\delta_{1}^{\left(t\right)}$ relative to the predicted advice accuracy or ${\hat{\mu }}_{1}^{\left(t\right)}$:

(8) $$\delta_{1}^{\left(t\right)}=u^{\left(t\right)}-{\hat{\mu }}_{1}^{\left(t\right)}$$

Subsequent precision‐weighted PE updates are hierarchically structured, necessitating the use of $\delta_{1}^{\left(t\right)}$ to refine the second‐level belief about adviser fidelity $x_{2}^{\left(t\right)}$. State $x_{2}^{\left(t\right)}$ is continuous and Gaussian‐distributed, thus defined by sufficient statistics $\mu_{2}^{\left(t\right)}$ (mean) and $\pi_{2}^{\left(t\right)}$ (precision, i.e., inverse variance). The belief update at the second level ${∆\mu }_{2}^{\left(t\right)}=\mu_{2}^{\left(t\right)}$ = $\mu_{2}^{\left(t\right)}-{\hat{\mu }}_{2}^{\left(t\right)}$ with ${\hat{\mu }}_{2}^{\left(t\right)}=\mu_{2}^{\left(t-1\right)}$ is driven by $\delta_{1}$ and modulated by $\pi_{2}$ (Mathys et al. 2011):

(9) $$\Delta \mu_{2}^{\left(t\right)}=\frac{1}{\pi_{2}^{\left(t\right)}}\delta_{1}^{\left(t\right)}$$

This adjustment leads to a volatility PE, $\delta_{2}^{\left(t\right)}$, brought on by $\Delta \mu_{2}^{\left(t\right)}$. At the third level, the agent's estimation of phasic log‐volatility of the adviser's fidelity $x_{3}^{\left(t\right)}$ is captured by the sufficient statistics, $\mu_{3}^{\left(t\right)}$ and $\pi_{3}^{\left(t\right)}$, with a similar pattern as the second level. The prediction update ${∆\mu }_{3}^{\left(t\right)}=\mu_{3}^{\left(t\right)}-{\hat{\mu }}_{3}^{\left(t\right)}$ with ${\hat{\mu }}_{3}^{\left(t\right)}=\mu_{3}^{\left(t-1\right)}$ is driven by $\delta_{2}$ and adjusted by $\pi_{3}$ (Mathys et al. 2011):

(10) $$\Delta \mu_{3}^{\left(t\right)}\propto \frac{1}{\pi_{3}^{\left(t\right)}}\delta_{2}^{\left(t\right)}$$

The fully expanded update equation at the third level is:

(11) $$\Delta \mu_{3}^{\left(t\right)}=\frac{1}{2}\frac{\gamma_{2}^{\left(t\right)}}{\pi_{3}^{\left(t\right)}}\delta_{2}^{\left(t\right)}$$

with auxiliary expected precision

(12) $$\gamma_{2}^{\left(t\right)}=\kappa v_{2}^{\left(t\right)}\pi_{2}^{\left(t\right)}$$

and the predicted environmental volatility (as a function of anticipated phasic log‐volatility of adviser fidelity, $\mu_{3}^{\left(t-1\right)}$):

(13) $$v_{2}^{\left(t\right)}=\exp\left(\kappa \mu_{3}^{\left(t-1\right)}+\omega \right)$$

Following these belief updates regarding the adviser's fidelity and its volatility, the agent can update the probability of the advice accuracy ${\hat{\mu }}_{1}^{\left(t+1\right)}$. This corresponds to the logistic sigmoid functions of the current adviser fidelity expectation:

(14) $${\hat{\mu }}_{1}^{\left(t+1\right)}=s\left(\mu_{2}^{\left(t\right)}\right)=\frac{1}{1+\exp\left(-\mu_{2}^{\left(t\right)}\right)}$$

With each new trial, a new cycle of these hierarchical updates is executed. Since these updates are precision‐ weighted PEs, our analysis encompasses the driving PEs ($\delta_{1}$ and $\delta_{2}$) and the precision weights ($\pi_{2}$ and $\pi_{3}$).

Two additional, simpler PEs can be derived from the task and the model, respectively, that relate directly to the observed outcomes. The cue PE ($\delta_{c}^{\left(t\right)}$) is calculated as the difference between the observed outcome $u^{\left(t\right)}$ and the cue $c^{\left(t\right)}$:

(15) $$\delta_{c}^{\left(t\right)}=u^{\left(t\right)}-c^{\left(t\right)}$$

Moreover, the outcome PE ($\delta_{b}^{\left(t\right)}$) is the discrepancy between the actual outcome $u^{\left(t\right)}$ and the predicted outcome $\mu_{b}^{\left(t\right)}$:

(16) $$\delta_{b}^{\left(t\right)}=u^{\left(t\right)}-\mu_{b}^{\left(t\right)}$$

where the predicted outcome is the weighted mean of advice‐based (${\hat{\mu }}_{1}^{\left(t\right)}$) and cue‐based (${\tilde{c}}^{\left(t\right)}$) probability (the probability indicated by the visual pie chart of the recommended color being correct):

(17) $$\mu_{b}^{\left(t\right)}=\zeta {\hat{\mu }}_{1}^{\left(t\right)}+\left(1-\zeta \right){\tilde{c}}^{\left(t\right)}$$

with *ζ* being the participant‐specific social (advice) weight parameter.

This results in a hierarchy of six PEs and precisions informing beliefs and their revisions. Sequentially, they are: (i) cue PE ${\tilde{\delta }}_{c}$, (ii) advice PE $\delta_{1}$, (iii) outcome PE $\delta_{b}$, (iv) precision of belief about advice fidelity $\pi_{2}$, (v) volatility PE $\delta_{2}$, and (vi) precision of belief about volatility $\pi_{3}$.

While the initial four regressors are computationally independent of each other, they all represent low‐level PEs and precisions, as illustrated by the equations for ${\tilde{\delta }}_{c}$, $\delta_{1}$, and $\delta_{b}$ above. The advice belief precision is given by:

(18) $$\pi_{2}^{\left(t\right)}=\frac{1}{\frac{1}{\pi_{2}^{\left(t-1\right)}}+v_{2}^{\left(t\right)}}+{\hat{\mu }}_{1}^{\left(t\right)}\left(1-{\hat{\mu }}_{1}^{\left(t\right)}\right)$$

The HGF model for our task suggests that these three low‐level PEs and the advice belief precision are calculated initially, prior to the computation of subsequent high‐level metrics in a strictly delineated sequence: $\delta_{2}$ is contingent upon $\pi_{2}$ directly and $\delta_{1}$ as it encompasses $\Delta \mu_{2}$:

(19) $$\delta_{2}^{\left(t\right)}=\left(\frac{{\hat{\pi }}_{2}^{\left(t\right)}}{\pi_{2}^{\left(t\right)}}\right)+{\left(\pi_{2}^{\left(t\right)}\right)}^{2}{\hat{\pi }}_{2}^{\left(t\right)}{\left(\Delta \mu_{2}^{\left(t\right)}\right)}^{2}-1$$

$\pi_{3}$ is influenced by $\delta_{2}$:

(20) $$\pi_{3}^{\left(t\right)}={\hat{\pi }}_{1}^{\left(t\right)}+\frac{1}{2}{\left(\gamma_{2}^{\left(t\right)}\right)}^{2}+{\left(\gamma_{2}^{\left(t\right)}\right)}^{2}\delta_{2}^{\left(t\right)}-\frac{1}{2}\gamma_{2}^{\left(t\right)}\delta_{2}^{\left(t\right)}$$

with the prediction precision for volatility defined by:

(21) $${\hat{\pi }}_{3}^{\left(t\right)}=\frac{1}{\frac{1}{\pi_{3}^{\left(k-1\right)}}+\theta }$$

This framework establishes a hierarchical computational structure where PEs and precisions are iteratively refined and transmitted to higher levels, thereby forecasting a temporal sequence of corresponding neurophysiological occurrences.

#### 2.4.4 Response Model

The response model describes how subjects' inferred states about the world guide their decisions to follow or disregard advice. It integrates both social and non‐social information sources: the social information corresponds to the participant's current belief (${\hat{\mu }}_{1}^{\left(t\right)}$) in the adviser's accuracy, while the non‐social cue ($c^{\left(t\right)}$) is the outcome probability indicated by the pie chart. Our response model assumes that participants decisions are based on a weighted average of these two sources, with *ζ* representing the weight given to the adviser's advice:

(22) $$b^{\left(t\right)}=\zeta {\hat{\mu }}_{1}^{\left(t\right)}+\left(1-\zeta \right)c^{\left(t\right)}$$

Finally, the probability that a participant will follow the advice (*y* = 1) is determined by a sigmoid function applied to the integrated belief *b*:

(23) $$p\left(y^{\left(t\right)}=1|b^{\left(t\right)}\right)=\frac{b^{\left(t\right)\beta }}{b^{\left(t\right)\beta }+{\left(1-b^{\left(t\right)}\right)}^{\beta }}$$

with

(24) $$\beta =\exp\left(-{\hat{\mu }}_{3}^{\left(t\right)}+\nu \right)$$

where *β* is the slope of the sigmoid and varies according to the estimated volatility of the adviser's intentions (${\hat{\mu }}_{3}^{\left(t\right)}$). The term *ν* represents decision noise (inverse decision temperature), which remains constant across trials and is independent of the estimated volatility (lower values indicate larger decision noise). This response model was consistently selected as the winning model in previous work (Diaconescu et al. 2014; Diaconescu, Litvak, et al. 2017), suggests that a reduction in the estimated volatility leads to more deterministic decisions based on advice validity, while higher volatility results in more exploratory behavior due to increased uncertainty about the adviser's intentions.

The models were implemented in Matlab (2023a) (https://www.mathworks.com) utilizing the HGF toolbox (version 3.0) from the TAPAS software suite (Frässle et al. 2021), which is available as open‐source code (https://github.com/translationalneuromodeling/tapas/releases/tag/v3.0.0). For the perceptual models, the ‘tapas_hgf_binary’ function was used for the standard 3‐level HGF, while the ‘tapas_hgf_ar1_binary’ function was employed for the mean‐reverting HGF.

#### 2.4.5 Bayesian Model Selection and Model Fitting

To compare the standard HGF and the mean‐reverting HGF, we used random‐effects Bayesian model selection (Stephan, Penny, et al. 2009; Rigoux et al. 2014) through the VBA toolbox (Daunizeau et al. 2014) (https://mbb‐team.github.io/VBA‐toolbox/). Additionally, we included two control models wherein all perceptual model parameters were fixed to those of an ideal Bayesian observer, optimized based solely on the inputs using the ‘tapas_bayes_optimal_binary’ function. The purpose of these control models was to evaluate if estimating perceptual model parameters is necessary for either of the main models. These ‘null’ models operate under the assumption that variations in advice‐following behavior are driven exclusively by response model parameters, namely the social bias and decision noise.

We report protected exceedance probabilities (PEP), which estimate the probability that one model best explains the data, accounting for the possibility that observed differences in exceedance probabilities could arise by chance (Stephan, Penny, et al. 2009; Rigoux et al. 2014). Furthermore, we provide relative model frequencies *f* as a measure of effect size, representing the probability that a given model is the most suitable for a randomly chosen participant. Priors for model parameters were based on Hauke et al. 2024, with details in Appendix S1.

#### 2.4.6 Model and Parameter Recovery

We assessed model and parameter recoverability through a series of simulations as done previously (Hauke et al. 2022, 2024) and conducted a parameter recovery analysis to estimate the upper bound of reliability. The mean‐reverting HGF emerged as the winning model (Figure S1) with both standard and mean‐reverting HGF showing good parameter recovery (Figure S2). These results held when additional control models were considered (Appendix S1).

### 2.5 EEG Recording and Preprocessing

EEG recordings were obtained using a 64‐electrode BioSemi MP150 System with active electrodes. Electrooculograms (EOGs) were captured using electrodes placed on the supraorbital and infraorbital ridges of the left eye and at the outer canthi of both eyes. The signals were digitized at 1024 Hz using a DC amplifier. EEG data was preprocessed and analyzed using SPM12 (http://www.fil.ion.ucl.ac.uk/spm/; version 7487) and MATLAB (R2024b version 24.2.0.2806996; https://www.mathworks.com). The EEG data was high‐pass filtered at 0.5 Hz, down‐sampled to 256 Hz, and low‐pass filtered at 30 Hz. The data was segmented into 750 ms epochs around outcome onsets (−100 to 650 ms), with baseline correction applied using a −100 to 0 ms pre‐stimulus baseline.

Eyeblink events were detected using a thresholding method applied to the vertical EOG channel and setting the eyeblink detection threshold to 3 standard deviations. The EEG data was then segmented into 1000 ms intervals, spanning from −500 to 500 ms around the identified eyeblink events. Signal space projection (SSP) implemented in SPM12 (Nolte and Hämäläinen 2001) was utilized for the detection and removal of the first eyeblink component from the EEG data. The effectiveness of the eyeblink correction was assessed by visually examining the average eyeblink event‐related potentials (ERPs) before and after eyeblink correction.

Following eyeblink correction, trials with amplitudes exceeding ±100 μV were discarded as artifacts. There were 166 [165, 170] (median [25th percentile, 75th percentile]) artifact‐free trials.

### 2.6 Single Trial EEG Analysis

Following Bayesian model comparison, we extracted the trajectories of the computational quantities from the winning mean‐reverting HGF model for each subject. We used these trajectories as explanatory variables in a general linear model (GLM) to investigate correlations between prediction errors (PEs) and precisions with EEG amplitude. Our aim was to explain the observed ERP responses at the single‐trial level across different channels and peristimulus time (PST). Single‐trial EEG data was converted into scalp images for all 64 channels using a voxel size of 4.3 mm × 5.4 mm × 2.0 ms, covering a post‐stimulus interval of 100–550 ms, and images were smoothed with a Gaussian kernel (full width at half maximum: 16 × 16 mm) to meet the assumptions of Gaussian random field theory (Worsley et al. 1996; Kiebel and Friston 2004).

At the individual subject level, we constructed GLMs with an intercept term and *z*‐standardized computational trajectories to analyze EEG amplitude variations across PST. We defined three separate GLMs incorporating multiple regressors capturing learning signals at different levels of the hierarchy assumed by the HGF: the first combined cue PE ($\delta_{c}^{\left(k\right)}$) and outcome PE ($\delta_{b}^{\left(k\right)}$); the second included absolute low‐level advice PE ($\delta_{1}^{\left(k\right)}$) and the corresponding advice precision ($\pi_{2}^{\left(k\right)}$); and the third included signed high‐level volatility PE ($\delta_{2}^{\left(k\right)}$) and the corresponding volatility precision ($\pi_{3}^{\left(k\right)}$). The results of signed low‐level advice PE ($\delta_{1}^{\left(k\right)}$) are provided in Appendix S1.

To assess potential collinearity, we computed subject‐level correlations between the two regressors within each GLM. Mean correlations (±SD) across participants were as follows: PEs GLM ($\delta_{c}^{\left(k\right)}$, $\delta_{b}^{\left(k\right)}$): 0.68 ± 0.23; lowPE GLM ($|\delta_{1}^{\left(k\right)}|$, $\pi_{2}^{\left(k\right)}$): 0.06 ± 0.05; highPE GLM ($\delta_{2}^{\left(k\right)}$, $\pi_{3}^{\left(k\right)}$): −0.07 ± 0.09. These values indicate moderate correlation between cue and outcome PEs, but only weak correlations between advice PE/precision and volatility PE/precision, suggesting that collinearity was not a major concern. For completeness, cue and outcome PEs were also examined in separate GLMs; sensor‐level results were comparable to those reported in the main text (Figure S3).

For each computational quantity, we conducted an *F*‐test at each point across the three‐dimensional sensor space (2D) × time volume to determine if the parameter estimates significantly differed from zero, creating beta images that were used for second‐level analysis.

For group‐level analysis, we examined the main effects of each computational trajectory, using the first‐level beta images as inputs. We identified significant effects through thresholded *F*‐statistical parametric maps (SPMs), both at the peak (*p*_p_ < 0.05) and cluster (*p*_c_ < 0.05) levels, applying family‐wise error (FWE) correction based on Gaussian random field theory, with a cluster defining threshold set at *p <* 0.001 uncorrected (Flandin and Friston 2019). This approach allowed us to determine when in time and where in sensor‐space precisions and PEs correlate with EEG amplitudes.

### 2.7 Phase Analysis

The task comprised 170 trials divided into three phases: a stable phase with consistently helpful advice (*n* = 34), a volatile phase where the adviser's intentions fluctuated rapidly (*n* = 102), and a second stable phase with consistently helpful advice (*n* = 34) (Figure 1B). The average ERP was computed across trials for each phase.

For each participant, phase‐specific ERP waveforms were converted into scalp images as outlined in the previous section. First‐level images were used as inputs for the second‐level GLM to assess the effect of phase‐specific waveform expression across sensor space and peristimulus time. The model included the phase variable with three levels (one for each phase). Significant effects were identified using the same threshold criteria described previously.

### 2.8 Source Analysis

Finally, we used multiple sparse priors (MSP) source reconstruction to estimate cortical source distributions responsible for the sensor‐level single‐trial EEG data (Friston et al. 2008). The sources were confined to 37 predefined cortical locations, informed by previous fMRI studies utilizing the same task (Diaconescu, Mathys, et al. 2017; Diaconescu, Litvak, et al. 2017) (refer to Table S1 for source coordinates). Source reconstruction was applied to both the outcome response waveform (phase analysis) and single‐trial EEG data (computational analysis) to estimate source time courses.

In the computational analysis, mirroring the sensor space analysis, we defined five GLMs. These included: cue and outcome PEs: $\delta_{c}^{\left(t\right)}$ and $\delta_{b}^{\left(t\right)}$; low‐level pwPE components: absolute $\delta_{1}^{\left(t\right)}$ and $\pi_{2}^{\left(t\right)}$; high‐level pwPE components: $\delta_{2}^{\left(t\right)}$ and $\pi_{3}^{\left(t\right)}$.

Each GLM included an intercept term and *z*‐standardized computational trajectories to account for changes in source amplitude across trials at the first level. The first‐level *β* waveforms represented the impact of each computational variable in explaining the absolute source amplitude, taking into account the effects of other regressors in the design matrices. These *β* waveforms were converted into images for second‐level analysis, allowing us to determine the timing of each computational variable's expression within trials. The same statistical thresholds used in the sensor analysis were applied here.

## 3 Results

### 3.1 Model Evaluation and Selection

The mean‐reverting HGF was identified as the winning model (Figure S1). Its protected exceedance probability PEP was 99.9%, and its relative model frequency (*f*), indicating the probability that a randomly selected participant would be best explained by this model, was *f* = 0.778.

The mean‐reverting HGF demonstrated good parameter recovery (Figure S2). Four of the seven parameters, including the decision noise *v* and drift‐equilibrium point *m*_3_, met the a priori recovery criterion in every simulation. Recovery for $\mu_{2}^{\left(0\right)}$, $\mu_{3}^{\left(0\right)}$, and $\kappa_{2}$ was moderate. Parameter interdependencies were low (|*r*| < 0.6), with the exception of a stronger correlation between $\kappa_{2}$ and $\omega_{2}$ (*r* = 0.77). Detailed correlations and average parameter estimates are provided in Appendix S1. We also examined the social bias parameter *ζ*, which reflects the relative weighting of advice versus non‐social cues, and found that participants on average showed a bias toward non‐social information (mean *ζ* = 0.428, SD = 0.150), with values below 0.5 indicating greater reliance on cues than advice (see Appendix S1). All subsequent model‐based results are derived from the mean‐reverting HGF.

### 3.2 Impact of Volatility on Outcome‐Related Responses

Next, we examined how the volatility manipulation affected ERP amplitudes following outcome presentation during three task phases: an initial stable phase (S1), a volatile phase (V), and a subsequent stable phase (S2).

**FIGURE 3:**
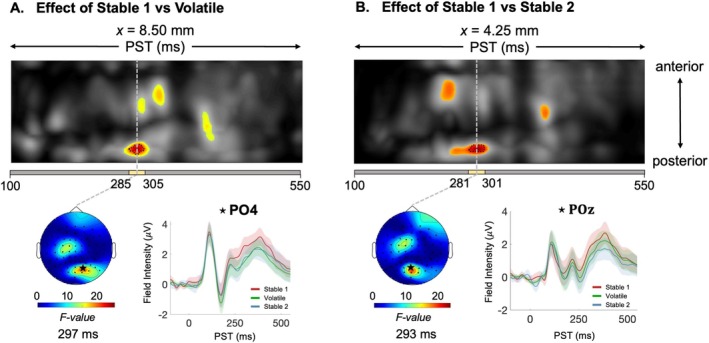
Effect of task phase on outcome response event‐related potentials (ERPs). Maximum intensity projections of F‐maps contrasting ERPs between (A) initial stable phase and volatile phase, and (B) the initial stable phase and final stable phase across anterior to posterior scalp locations (top), spanning 100–550 ms post‐stimulus. Significant peak‐level effects (*p* < 0.05, whole‐volume FWE‐corrected) are outlined with black contours, and regions where *F*‐values exceed the cluster‐defining threshold (*p* < 0.05, corrected) are shown in color. A yellow bar beneath each t‐map indicates the duration of significant peak effects, from the earliest to the latest significant time points. On the left, scalp maps illustrating the peak effect of the identified cluster at the peak peri‐stimulus time (PST) on a 2D sensor layout are shown. Outcome‐related ERPs, averaged for each phase across the electrode at the peak of the significant cluster, are displayed, with the selected electrode marked with a star on the scalp map. Note that we see additional clusters which pass an uncorrected threshold of *p* < 0.001 but do not remain significant after multiple‐comparison correction.

Using *F*‐tests, we performed pairwise comparisons among these phases (S1 vs. V, S1 vs. S2, and V vs. S2) (Table S2). For the S1 versus V comparison, we identified a significant peak‐level effect at 297 ms in parietal–occipital electrodes (*F*_(1,84)_ = 25.81; *p* = 0.010; Figure 3A) and a significant cluster‐level effect at 328 ms in frontal electrodes (*F*_(1,84)_ = 18.15; *p* = 0.033). In the S1 versus S2 comparison, we found a significant peak‐level effect at 293 ms in parietal–occipital electrodes (*F*_(1,84)_ = 23.99; *p* = 0.018; Figure 3B) and a significant cluster‐level effect at 250 ms in frontal electrodes (*F*_(1,84)_ = 19.18; *p* = 0.024). No significant differences occurred for the V versus S2 comparison.

**FIGURE 4:**
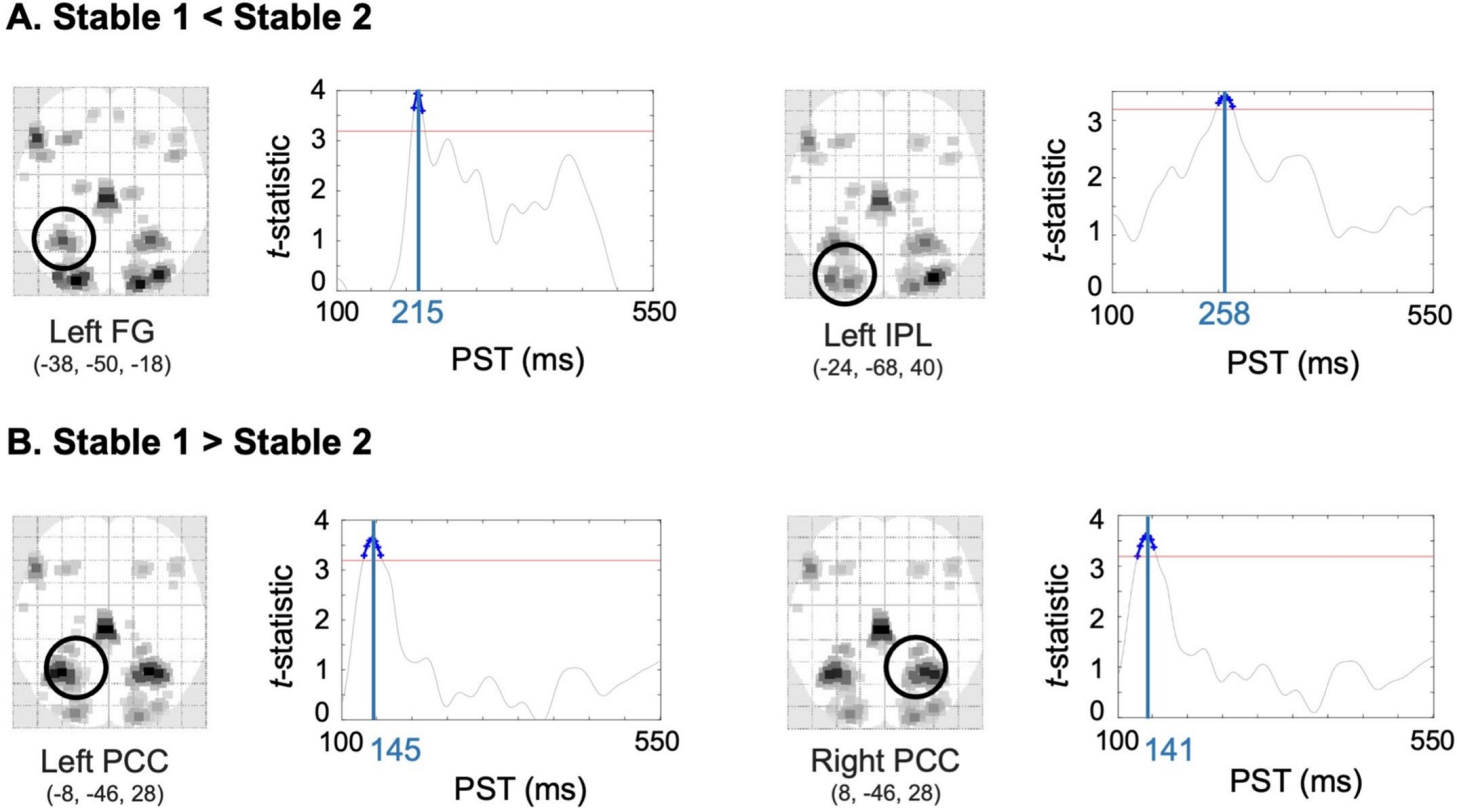
Cortical sources of outcome‐related task phase response. (A) Source activation of outcome‐related event‐related potentials (ERPs) at their peak time points, visualized on an SPM‐glass brain in neurological orientation. Significant task phase effect are presented for the left fusiform gyrus (FG) and left inferior parietal lobe (IPL) across peri‐stimulus time (PST). The significance threshold, set under peak‐family wise error (FWE) correction, is represented by a red horizontal line. All time points exceeding this threshold are shown in blue, with the peak time point identified by a blue vertical line. (B) Source activation of outcome‐related ERPs is shown for the left and right posterior cingulate cortex (PCC), peaking at 145 and 141 ms post‐stimulus, respectively.

Cortical source analysis of these sensor‐level phase effects revealed differential activation patterns between phases. Specifically, the S1 phase exhibited reduced amplitude compared to the S2 phase in the left fusiform gyrus (FG) (*t*_(1,84)_ = 3.73, *p* = 0.004) and left inferior parietal lobule (IPL) (*t*_(1,84)_ = 3.42, *p* = 0.008), with peaks at 215 and 258 ms, respectively (Figure 4A; Table S4). In contrast, bilateral posterior cingulate cortex (PCC) regions showed increased amplitude during S1 (left PCC: *t*_(1,84)_ = 3.62, *p* = 0.006, peak 145 ms; right PCC: *t*_(1,84)_ = 3.60, *p* = 0.006, peak 141 ms) (Figure 4B). Additionally, the right primary visual cortex (V1) showed greater amplitude during S1 compared to V at 266 ms (*t*_(1,84)_ = 3.22, *p* = 0.017). No significant differences were observed for the V versus S2 comparison.

### 3.3 Computational Model Trajectories and EEG Correlates

By utilizing the HGF framework to analyze the single‐trial EEG data, we obtain a specific computational structure, where PEs and precisions are progressively refined and transmitted upward through the hierarchy.

**FIGURE 5:**
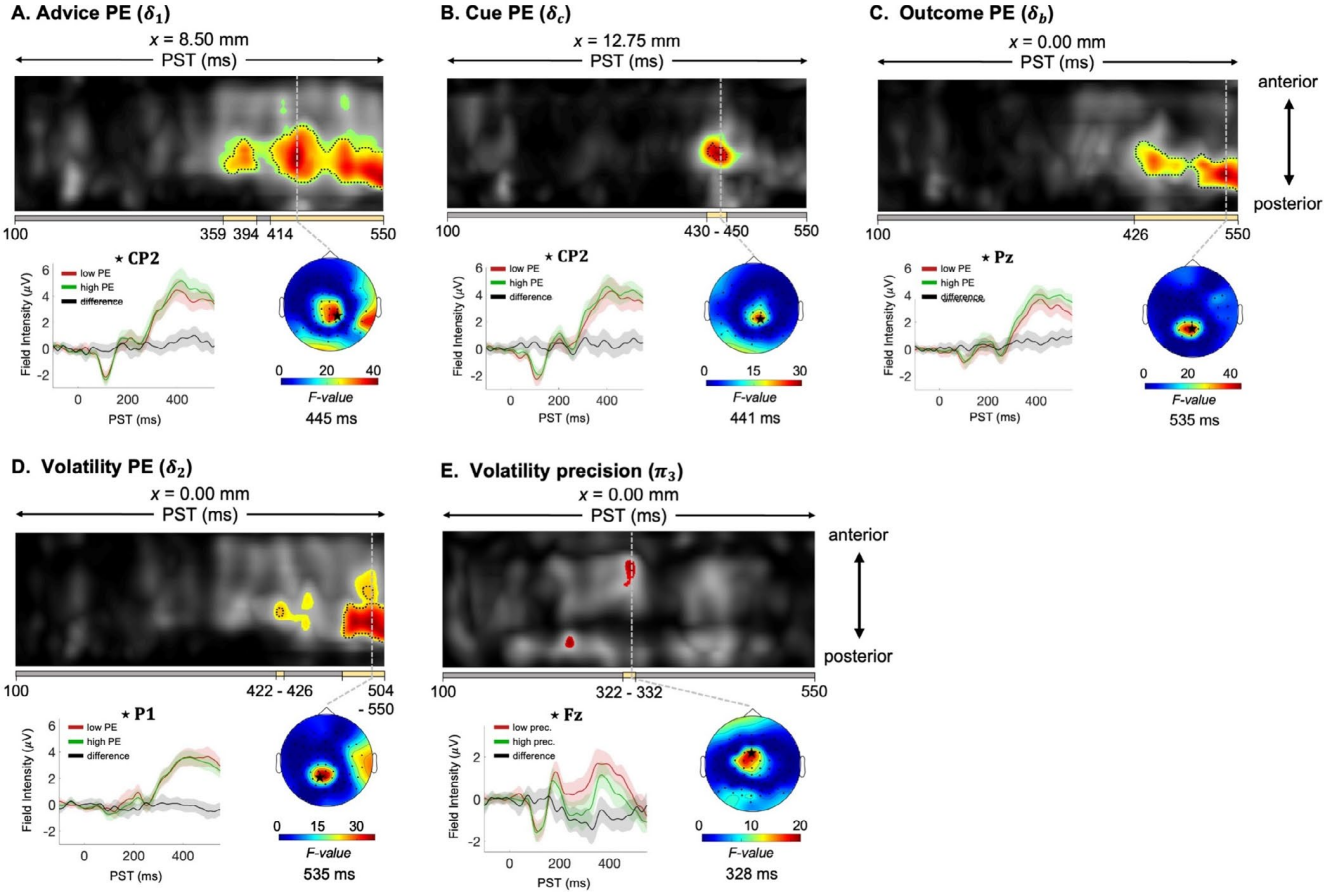
Impact of model trajectories on outcome response. (A–E) Maximum intensity projections of F‐maps across anterior to posterior scalp locations (top), spanning 100–550 ms post‐stimulus. Significant peak‐level effects (*p* < 0.05, whole‐volume FWE‐corrected) are outlined with black contours, and regions where *F*‐values exceed the cluster‐defining threshold (*p* < 0.05, corrected) are shown in color. A yellow bar beneath each F‐map indicates the temporal extent of significant clusters, from the earliest to the latest significant time points. On the left, ERPs averaged across the electrode at the peak of the significant cluster, calculated using the top and bottom 10% of PE (or precision) values, are shown. The selected electrode is marked with a star on the scalp map (right), which illustrates the peak effect of the identified cluster at the peak peri‐stimulus time (PST) on a 2D sensor layout.

We observed significant effects of advice PE from 352 to 550 ms post‐stimulus, peaking at 445 ms ($\delta_{1}$, *F*_42_ = 41.49, *p* = 0.001), cue PE from 418 to 473 ms post‐stimulus, peaking at 441 ms ($\delta_{c}$, *F*_42_ = 29.30, *p* = 0.015), and outcome PE from 426 to 550 ms post‐stimulus, peaking at 535 ms ($\delta_{b}$, *F*_42_ = 44.68, *p* < 0.001), in central‐parietal electrodes (Figure 5A–C). No significant effects were detected for advice precision ($\pi_{2}$).

Moving up the hierarchy, the volatility PE ($\delta_{2}$) was expressed from 500 to 550 ms post‐stimulus, with a maximal peak at 535 ms (*F*_42_ = 36.42, *p* = 0.003; Figure 5D) in parietal electrodes. A significant cluster‐level effect for volatility precision ($\pi_{3}$) was observed from 320 to 332 ms in frontal electrodes (cluster, *F*_42_ = 19.17; *p* = 0.047; Figure 5E).

Source analysis of the model regressors revealed no significant *F*‐test effects. Although some effects were observed using one‐sample *t*‐tests, these should be interpreted with caution given the absence of significant *F*‐test findings (Table S6).

### 3.4 Relationship With Functional Outcomes

We investigated the relationship between outcome‐related responses and baseline psychosocial functioning, focusing on correlations in both sensor and source space, and identified significant correlations with psychosocial function primarily in source space.

#### 3.4.1 Correlations Between Outcome‐Related Responses and Function

No significant correlations were found between baseline psychosocial functioning and outcome‐related responses in sensor space. Similarly, examining task phase difference waveforms—comparing the volatile phase to the first stable phase and the second stable phase to the first—revealed no significant associations in sensor space. However, we found a positive correlation between role functioning and the difference waveform comparing the second stable phase and volatile phase at 242 ms in frontal electrodes (*t*_(1,41)_ = 4.81, *p* = 0.040).

**FIGURE 6:**
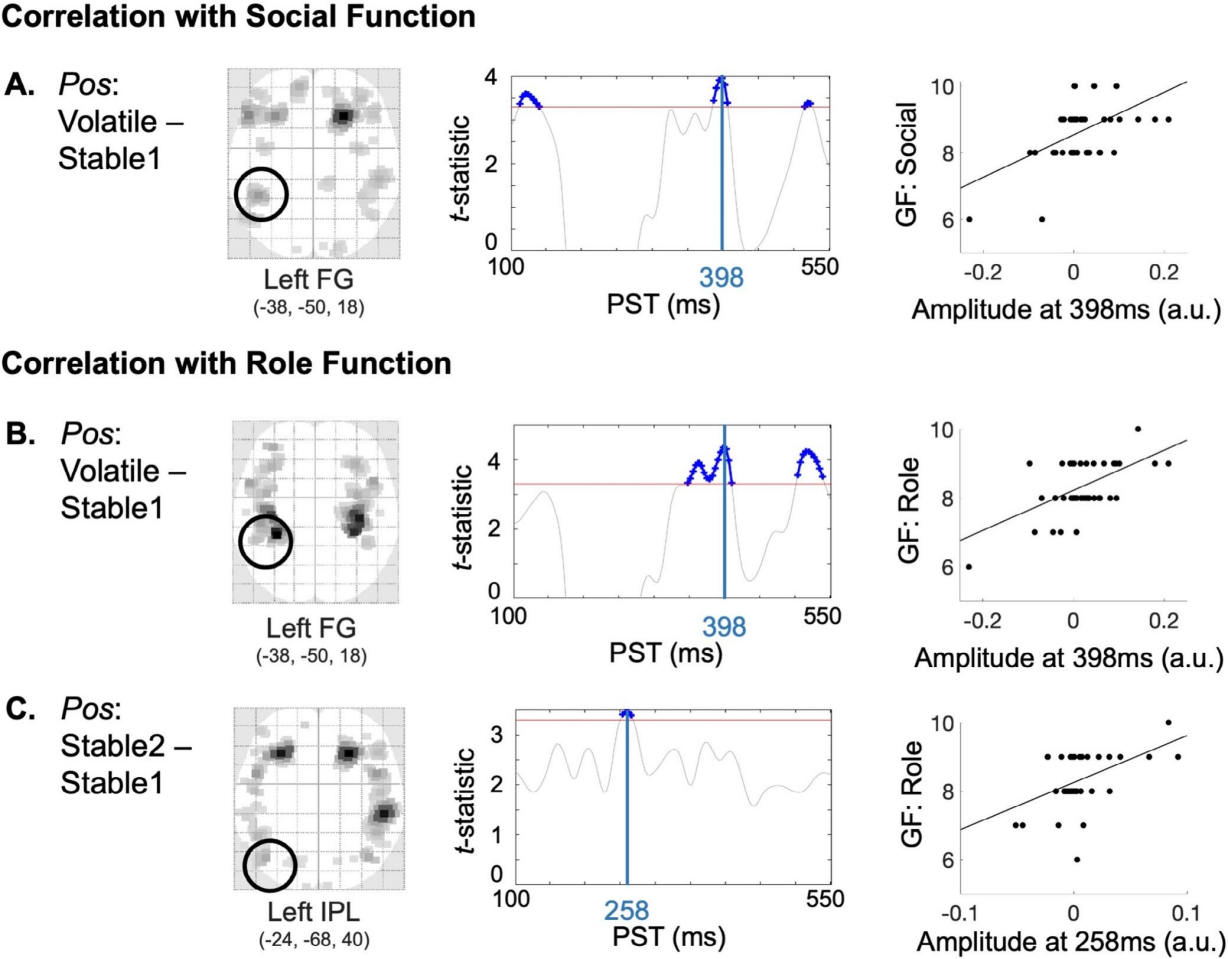
Cortical sources for outcome‐related response and functioning. (A) A negative correlation between the grand‐averaged phase difference waveform (volatile phase—initial stable phase) and social function, with a maximal peak at 398 ms in the left fusiform gyrus (FG). Source activations are depicted on an SPM‐glass brain in neurological orientation (left). Significant t‐contrasts between the outcome difference waveform and global function (GF) scores over peri‐stimulus time (PST) are displayed in the middle. A red horizontal line indicates the significance threshold under peak‐family wise error (FWE) correction, with all surpassing time points in blue. The peak time point is highlighted by a blue vertical line. The scatter plot (right) displays the correlation between GF scores and the amplitude of the outcome difference waveform at the peak time point. (B) Source activation of grand‐averaged phase difference waveform (volatile phase—first stable phase) and role function, peaking at 398 ms in the left fusiform gyrus (FG). (C) Source activation of grand‐averaged phase difference waveform (second stable phase—first stable phase) and role function, peaking at 258 ms in the left inferior parietal lobe (IPL).

Source‐space analyses revealed additional early negative correlations in the difference waveform when comparing the volatile phase to the initial stable phase (Table S7). Specifically, the difference waveform amplitude in the left FG correlated positively with social functioning (*t*_(1,41)_ = 3.95, *p* = 0.004) and role functioning (*t*_(1,41)_ = 4.40, *p* = 0.001) at 398 ms post‐stimulus (Figure 6A,B). Additionally, for the difference waveform comparing the second stable phase to the initial stable phase, significant correlations were found between activity in the left IPL and role functioning (*t*_(1,41)_ = 3.47, *p* = 0.011; Figure 6C). Additional correlations were found between the outcome‐related ERPs and activity in the left V1 and right middle cingulate cortex (MCC). However, these correlations were driven by an outlier with low function scores and extreme beta values. After excluding this subject, these correlations were no longer significant (Table S4). Finally, additional effects were observed in the difference waveform between the second stable phase and volatile phase; in the interest of brevity, these findings are summarized in Table S7.

#### 3.4.2 Model‐Based Correlations in Sensor Space

**FIGURE 7:**
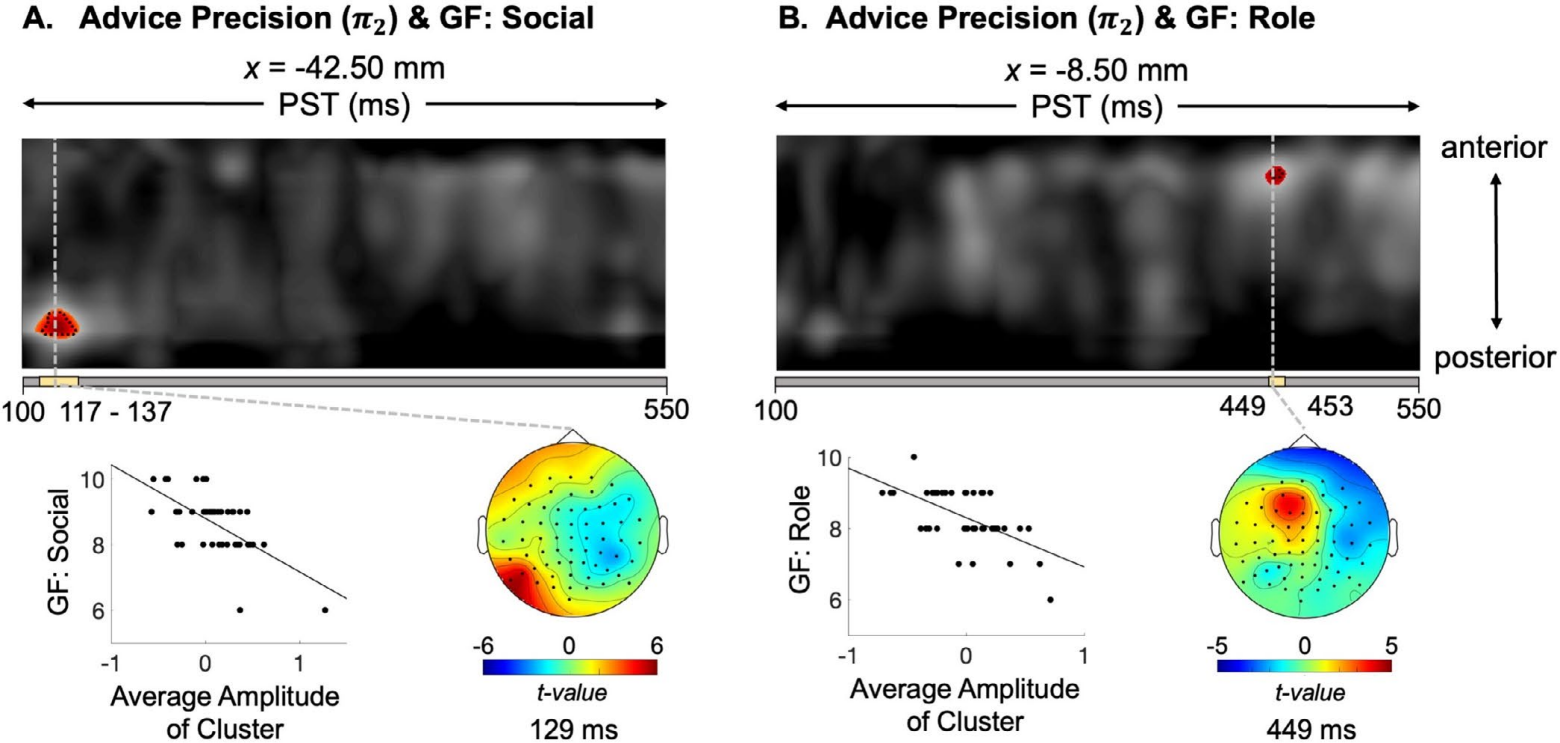
Negative correlation of advice precision with function. (A, B) Maximum intensity projections of t‐map illustrating the correlation between global function (GF) and advice precision across and anterior to posterior scalp locations, spanning 100–550 ms post‐stimulus. (Top) Significant peak‐level effects (*p* < 0.05, whole‐volume FWE‐corrected) are outlined with black contours, and regions where F‐values exceed the cluster‐defining threshold (*p* < 0.05, corrected) are shown in colour. A yellow bar beneath each F‐map indicates the temporal extent of significant clusters, from the earliest to the latest significant time points. On the left, the scatter plot shows the relationship between GF scores and the first‐level *β* estimates at the peak coordinate. On the right, the scalp map displays the peak effect of the given cluster at the peak peri‐stimulus time (PST) on a 2D sensor layout.

Although no group‐level effects were found for advice precision ($\pi_{2}$) in sensor space, we observed significant negative correlations between the precision effect and GF: Social at 129 ms in temporal electrodes (*t*_41_ = 5.4, *p* = 0.009) and GF: Role at 449 ms in frontocentral electrodes (*t*_41_ = 4.79, *p* = 0.041) (Figure 7A,B; Table S8). An *F*‐test further confirmed that the magnitude of the advice precision effect correlated with GF: Social at 129 ms (*F*_41_ = 28.92, *p* = 0.017). The GF: Role correlation did not remain significant.

Since the data were collected from an early psychosis study (Hauke et al. 2024), the HC sample included elevated rates of cannabis use, and supplementary covariate analyses revealed one significant effect for volatility PE (*δ*_2_) at 434 ms (*F*_41_ = 34.35, *p* = 0.005), with no other significant effects observed (see Appendix S1).

#### 3.4.3 Model‐Based Correlations in Source Space

Source‐space analyses revealed associations between functioning and computational update quantities in several visual processing regions, including the cuneus (CC), LG, inferior occipital gyrus (IOG), and fusiform gyrus (FG), as well as the posterior cingulate cortex (PCC). Full results are provided in Tables S9 and S10; below we report effects for the FG and PCC.

In the social domain, the right PCC showed a positive correlation with advice PE expression ($\delta_{1}$), peaking at 207 ms (*t*_41_ = 3.30, *p* = 0.017). In the role domain, a negative correlation was found with the left FG, peaking at 430 ms (*t*_41_ = 3.94, *p* = 0.002), while both left (*t*_41_ = 4.18, *p* = 0.002) and right (*t*_41_ = 4.30, *p* = 0.001) PCC positively correlated with advice PEs, peaking at 379.

Additionally, the left FG and PCC correlated with the volatility PE effect ($\delta_{2}$) across the social and role domains. In the social domain, peak activity was found at 422 ms in the left FG (*t*_41_ = 3.42, *p* = 0.008); in the left PCC, peaks emerged at 312 ms (*t*_41_ = 3.47, *p* = 0.011) and 430 ms (*t*_41_ = 3.39, *p* = 0.013); and in the right PCC, peaks were noted at 309 ms (*t*_41_ = 3.54, *p* = 0.009) and 426 ms (*t*_41_ = 3.56, *p* = 0.009). In the role domain, peak activations occurred at 430 ms in the left FG (*t*_41_ = 4.21, *p* = 0.001) and 371 ms in the left PCC (*t*_41_ = 3.46, *p* = 0.011).

#### 3.4.4 Correlations Between Parameters Estimates and Function

Finally, we examined associations between parameter estimates and baseline functioning using Kendall's Tau correlation coefficient. Parameter estimates, single values obtained for each individual by fitting the model to behavioral data, independent of EEG measures, were correlated with baseline functioning. A significant positive correlation was observed between social functioning and the inverse decision temperature parameter *ν* (*r*_*τ*_ = 0.246, *p* = 0.042).

## 4 Discussion

This study aimed to clarify how hierarchical Bayesian processes shape adaptive learning during social interactions, especially when faced with uncertainty regarding others' intentions. We advanced understanding of social learning under volatility through three key findings: First, neural responses significantly varied between stable and volatile task phases, potentially reflecting heightened sensitivity to uncertainty. Second, our EEG analyses demonstrated an alignment between the temporal sequence of neural activity and the predicted hierarchical computations from the HGF model. Third, variations in source‐level EEG activity correlated with psychosocial functioning, highlighting clinically relevant neural markers that link neural processing and psychosocial outcomes.

### 4.1 Neural Correlates of Social Learning Under Volatility

In our sensor‐level phase analysis, EEG responses varied significantly across task phases, with increased posterior electrode activity during volatile phases, suggesting heightened sensitivity to uncertainty during volatile social contexts. The frontal effects we observed at 250–328 ms overlap in latency and topography with the canonical feedback‐related negativity (FRN) component (Falkenstein et al. 1991; Miltner et al. 1997; Gehring and Willoughby 2002), whereas parietal‐occipital effects fell outside the FRN's typical distribution (Figure 3). Furthermore, the heightened neural response extending into the second stable phase likely indicates a persistent perception of volatility, rather than recognition of a separate and distinct stable phase, possibly due to the short duration of this phase. This aligns with our modeling results, where the average participant drift equilibrium point (*m*_3_) exceeded the prior, suggesting a shift towards increased volatility perception over time (Table S12). Moreover, prior research has demonstrated that even a single ‘dose’ of volatility can have lasting effects on belief updating, persisting beyond a volatile phase (Mathys et al. 2011).

The FG, important for complex visual tasks like facial processing (Weiner and Zilles 2016), and the IPL, involved in both lower‐level spatial attention and higher‐level social cognition (Numssen et al. 2021), showed increased activity in the second stable phase compared to the initial stable phase (Figure 4A). Activity in the left FG during the volatile phase correlated positively with social and role functioning, while activity in the left IPL during the second stable phase correlated positively with role function. This may suggest that high‐functioning individuals may rely more on processing facial expressions or social signals in uncertain conditions. Given previous reports of FG hypoactivity in social–emotional disorders (Abdi and Sharma 2004; Seiferth et al. 2009; Li et al. 2010; Perlman et al. 2011), our findings underscore FG's role in adaptive social cognition.

Conversely, the PCC, which plays a role in attentional focus, showed reduced activity during the second stable phase. This aligns with existing literature indicating that the activity of the PCC diminishes when intense external focus is required (Leech and Sharp 2014). Such a response emphasizes the brain's ability to adapt to shifting attentional demands, particularly in situations where external references are needed to interpret unreliable social cues.

Taken together, these results highlight posterior regions such as FG and PCC in adaptive responses to social uncertainty. At the same time, other studies have emphasized the roles of the insula and prefrontal cortex (PFC) in uncertainty and salience processing. For example, recent intracranial EEG work has demonstrated insula and dorsomedial PFC involvement during a reward‐based learning task (Hoy et al. 2023), a lottery‐like probabilistic prediction task (Haufler et al. 2022), memory recall tasks (Das and Menon 2024), resting state (Das et al. 2022), and auditory deviance detection (Blenkmann et al. 2019). These findings underscore that multiple circuits contribute to uncertainty processing, with the precise network engagement likely depending on task demands. Our study focused on social inference, where participants judged an adviser's reliability using both video and probabilistic cues, a context that may preferentially recruit posterior regions supporting social cognition and context evaluation.

### 4.2 Temporal Dynamics of Hierarchical Bayesian Inference

Our EEG findings provide evidence supporting the hierarchical sequence of computations predicted by the HGF model (Diaconescu, Mathys, et al. 2017), demonstrating distinct temporal signatures for lower‐level (advice) and higher‐level (volatility) computations. These findings emphasize that social inference is a hierarchical process, in which learning from advice unfolds under a higher‐level representation of the adviser's intentions (Diaconescu et al. 2014; Diaconescu, Litvak, et al. 2017).

However, we observed weaker effects for the two hierarchically‐coupled precisions, and volatility precision ($\pi_{3}$) showed an earlier‐than‐expected onset. One possibility is that these early precision signals reflect neural adaptation (or repetition suppression), whereby a sensory population becomes less responsive to repeated inputs, as seen in standard‐tone mismatch‐negativity (MMN) paradigms (Näätänen et al. 2007; Garrido et al. 2009). Adaptation would manifest almost instantaneously upon stimulus presentation—consistent with our precision onset (~129 ms) and the early advice–function correlations—whereas prediction errors (PEs) emerge later as “messages” passed between distinct neuronal populations. Alternatively, the brain may utilize parallel processing for precisions and prediction errors, or perhaps utilizes different computational algorithms, like belief propagation (Jardri and Denève 2013). Further research should determine whether similar hierarchies exist in other learning contexts.

Although the task targets volatility while attempting to hold stochasticity approximately constant, these sources of uncertainty are not fully separable at the observation level, especially with binary outcomes. Moreover, because the advice streams originate from human advisers, within‐policy stochasticity may vary modestly across sequences, precluding orthogonal manipulation of volatility and stochasticity. Prior work suggests that apparent misestimation of volatility may in fact reflect altered learning about stochasticity (Piray and Daw 2021), and more recent evidence indicates that humans are capable of learning about both factors simultaneously (Piray and Daw 2024). We therefore interpret volatility‐related findings with this constraint in mind.

### 4.3 Functional Correlations and Clinical Implications

#### 4.3.1 Correlations Between Model Parameters and Functional Impairment

We observed significant correlations in source space between model parameters and functional impairment, particularly within the visual processing pathway, including the primary visual and association cortices, particularly the CC, LG, IOG, and FG, as well as the PCC. Connections from the LG and IOG feed into specific areas of the FG, such as the ventromedial visual area, which integrates color, form, and texture information for holistic recognition, and the fusiform face area, critical for processing facial features (Palejwala et al. 2020, 2021). Interestingly, significant correlations were observed exclusively in the left FG. Prior studies have highlighted functional differences between the left and right FG in face processing: the left FG is more involved in identifying face‐like features and assessing physical similarity, while the right FG categorizes these features as a face (Meng et al. 2012; Ma and Han 2012). These results suggest that functional impairments may be more closely related to non‐verbal social cues, such as changes in gaze or facial expression (Meng et al. 2012).

#### 4.3.2 Sensitivity to Advice Prediction Errors and Role Functioning

Activity in the left FG exhibited a negative correlation with advice PEs and baseline role functioning. This indicates that individuals with lower role functioning may respond differently to advice, whether the error is unintentional or deliberate. For these individuals, the discrepancy between expected and actual advice accuracy may appear more salient, prompting larger belief updates about the adviser's intentions. While such heightened sensitivity to adviser mistakes aligns with the aberrant salience hypothesis, often described in the early stages of psychosis where irrelevant sensory stimuli are given undue significance (Kapur 2003; Howes et al. 2020), it could also be adaptive in environments where conditions are changing. However, in stable environments, excessive learning from prediction errors may lead to maladaptive behaviors, as the frequent updating of beliefs can cause an enhanced perception of volatility during social exchange (Hauke et al. 2024; Powers et al. 2025).

#### 4.3.3 Role of PCC Activity in Social Learning and Psychosocial Functioning

The PCC demonstrated a positive correlation with advice PEs and psychosocial functioning, suggesting its central role in adaptive social learning. As a central component of the default mode network (DMN), the PCC is involved in arousal, awareness, internal and external attention, and detecting environmental changes (Leech and Sharp 2014). Its increased activity might reflect a focus on internal processes, such as memory retrieval or future planning. Individuals with high social functioning might rely more on these internal processes to assess whether advisers' mistakes are intentional or not, guiding more nuanced belief updates about the adviser's reliability.

Interestingly, the PCC also showed positive correlations with volatility PEs and psychosocial functioning. Volatility PEs represent adjustments to beliefs about the rate of change in an adviser's reliability. The ability to rapidly update these beliefs is particularly advantageous in unpredictable environments, where adaptability is critical. This enhanced adaptability likely involves integrating external cues, such as facial expressions, with internal cognitive processes through the coordinated activation of the FG and PCC. Additionally, the PCC has been implicated in signaling unexpected uncertainty (Payzan‐LeNestour et al. 2013), when an individual encounters deviations from expected patterns, it may contribute to recalibrating beliefs about an adviser's reliability. By responding to unexpected changes, the PCC likely helps individuals distinguish between transient inconsistencies and genuine shifts in trustworthiness, reinforcing its role in flexible social learning.

Structural and functional abnormalities in the PCC have been consistently observed across various clinical populations (Leech and Sharp 2014), including in autism spectrum disorders and schizophrenia (Barlati et al. 2020; Kozhuharova et al. 2020). These findings highlight the potential significance of PCC dynamics in emotional processing and theory of mind (TOM) tasks. Although the directionality of PCC activity in these disorders remains mixed, our results suggest that enhanced PCC engagement may facilitate more effective internal reasoning and decision‐making during social learning tasks.

Examining cortical computational hierarchies with EEG may prove useful for clinical purposes. Aberrant hierarchical Bayesian inference has been implicated in the pathophysiology of schizophrenia (Fletcher and Frith 2009; Stephan, Friston, et al. 2009; Adams et al. 2013; Powers et al. 2017, 2025) and autism (Lawson et al. 2014; Sevgi et al. 2016). Methods like single‐trial EEG analysis could complement fMRI by revealing the precise temporal order of computations and, crucially, by potentially uncovering distinct biological mechanisms for precision versus prediction‐error signals. If, as suggested above, precisions arise via near‐instantaneous neural adaptation while prediction errors reflect inter‐population message‐passing, then EEG's millisecond resolution offers insights that fMRI alone cannot.

Importantly, model‐based EEG allows us to test whether the brain operates according to a specific computational algorithm or whether alternative models are needed to better explain neural dynamics. This approach also opens avenues for investigating the effects of temporally sensitive treatments. For example, transcranial magnetic stimulation (TMS) or direct current stimulation (tDCS) could be timed to target high‐level prediction‐error processing windows without affecting earlier precision computations (or vice versa). By mapping and manipulating these time‐locked computations, model‐based EEG stands to refine both our mechanistic understanding and our evaluation of temporally sensitive interventions in psychiatric and neurotypical populations alike.

### 4.4 Limitations

Our study has several limitations. First, the experimental design did not fully capture the recursive nature of social inference (FeldmanHall and Nassar 2021). The advice was conveyed via pre‐recorded videos from past adviser‐player interactions (Diaconescu et al. 2014), which may have restricted social cognition to basic theory of mind inference (i.e., inferring the advisers' mental states) (Devaine et al. 2014a,2014b). This confines our conclusions to a specific level of social inference and omits the broader spectrum of theory of mind. However, it also standardizes the reasoning level among participants, allowing for the application of models like the HGF, which do not account for the recursive nature of social interactions (Diaconescu et al. 2014).

Second, although the task primarily targeted volatility while aiming to hold stochasticity constant (privileged adviser information fixed at 80%), advice validity nonetheless varied more widely in the volatile than in the stable phase. This asymmetry, stemming from the replayed human‐advice provenance (Diaconescu et al. 2014), limited a clean dissociation between policy change (volatility) and within‐policy consistency (stochasticity), especially with binary outcomes. Consequently, some effects may partly reflect learning about noise rather than volatility alone, and HGF parameters may capture variance arising from both sources. We therefore interpret volatility‐related findings with this constraint in mind, and note that stronger dissociation would require designs that orthogonally manipulate mean helpfulness and hazard rate of policy shifts (e.g., synthetic advice sequences or continuous outcomes). In addition, while the HGF framework aims to capture how volatility modulates the reliability of advice, it does not explicitly represent trustworthiness as a latent construct; representational models that approximate the probability of adviser trustworthiness may therefore provide a valuable complementary approach (Barnby et al. 2023).

Third, source localization in our study is limited by the spatial resolution of EEG. While EEG provides valuable temporal precision, its ability to localize activity to specific brain regions is inherently constrained. To mitigate this limitation, we incorporated spatial priors from an earlier fMRI study using the same task paradigm (Diaconescu, Litvak, et al. 2017), thereby enhancing the anatomical validity of our source estimates.

Finally, the data was collected from an early psychosis study (Hauke et al. 2024), using a functioning assessment not designed for broader populations (Cornblatt et al. 2007). Although our findings suggest significant links between social learning under volatility and psychosocial functioning, future research should employ more comprehensive functioning assessments, like WHODAS 2.0 (Gold 2014), to fully capture these effects.

A further consideration that may impact the interpretation of these results is the reliance on statistical thresholding methods to ascertain a temporal hierarchy. Within trial‐wise EEG analysis, the establishment of a hierarchy was determined through the sequencing of significant peri‐stimulus time points as identified within the SPM of the *F*‐statistic reporting PE‐ (or precision) effects on sensor space voxels (or channels). More precisely, the initial PST found within the first significant cluster, adjusted for FWE, for each computational measure was used as a benchmark for ordering. It should be emphasized, though, that the identified temporal hierarchy of the low‐ and high‐level PEs is robust; it remains consistent regardless of the statistical correction approach employed, be it at the peak or cluster level, and is also independent of the chosen criterion, whether it is the peak or the first significant voxel in the primary significant cluster.

In summary, this study advances our understanding of hierarchical Bayesian mechanisms underlying social learning and the temporal dynamics of neural computations that drive adaptive social inference. By linking computational model parameters to neural activity and psychosocial functioning, we reveal the importance of the FG and PCC in adaptive social responses. These findings not only provide a benchmark for studying theory of mind deficits in psychiatric populations, such as schizophrenia and autism, but also highlight the potential for computational modeling and single‐trial EEG analysis to serve as sensitive tools for probing hierarchical inference disruptions.

## Author Contributions

C.E.C. performed the data analysis and drafted the manuscript. D.J.H. conceptualized the study, recruited participants, collected data, contributed to the development of the analysis pipelines, and revised the manuscript. V.L. developed the EEG analysis pipelined used in the study and revised the manuscript. M.W. recruited participants, collected data, and revised the manuscript. C.A. provided rater training, supervision, and revised the manuscript. R.B. recruited participants, collected data, and revised the manuscript. S.B. and V.R. provided infrastructure, supervision, and revised the manuscript. A.O.D. conceptualized and supervised the study, wrote, and revised the manuscript.

## Funding

This work was supported by the Swiss National Science Foundation (Doc.Mobility, 200054 to D.J.H.; Ambizione, PZ00P3 167952 to A.O.D., Project grant: CRSK‐3 190834 to R.B.) and the Krembil Foundation (to A.O.D.).

## Conflicts of Interest

The authors declare no conflicts of interest.

## Supporting information


**APPENDIX S1:** Supporting information.

## Data Availability

The analysis code for this study is publicly available at https://github.com/colleenc11/COMPI_IOIO. Data will be made available under https://osf.io/4zf5q/overview upon acceptance of this manuscript.
