# Supplementary material for: Neural Dynamics of Social Cognition: A Single‐Trial Computational Analysis of Learning Under Uncertainty
Source: Hum Brain Mapp. 2026 Jan 14;47(1):e70433. doi: 10.1002/hbm.70433 (PMC12800744; doi:10.1002/hbm.70433)
Supplement: Supplementary file 1 — APPENDIX S1: Supporting information. [file HBM-47-e70433-s001.docx]

SUPPLEMENTARY INFORMATION

Neural Dynamics of Social Cognition: A Single-trial Computational Analysis of Learning under Uncertainty: Supplementary

Charlton *et al.*

# Supplementary

## *Inclusion and Exclusion Criteria*

Participants were eligible if they were 15 years of age or older. The exclusion criteria included: previous psychotic episodes, psychotic symptomatology secondary to an organic disorder, any past or present neurological disorder, a premorbid IQ below 70, assessed using the Mehrfachwahl-Wortschatz-Test, Version A ([Lehlr et al., 1995),](#Lehrl1995) color-blindness, substance use disorders as defined by the ICD-10 (except cannabis), consumption of alcohol or cannabis within the 24 hours preceding the tests, and regular drug consumption other than alcohol, nicotine, and cannabis. Drug consumption was initially checked during the admission interview and subsequently confirmed via a drug urine test before the EEG measurement. In cases of positive results participants were excluded or the assessments postponed until a negative result was obtained.

## *Source Coordinates*

The source coordinates for multiple sparse priors (MSP) source reconstruction are shown in Table S1.

## *Model Evaluation and Selection*

To assess the recoverability of our models and parameters, we conducted simulations as described previously ([Hauke et al., 2022](#SupHauke2022); [Hauke et al., 2024](#SupHauke2024)). This process involved creating 20 synthetic datasets for each of the 43 participants, using empirical parameter estimates derived from fitting all models to the actual participant data. Simulations were initialized with different random seeds to account for stochasticity. We then re-inverted the models on these synthetic datasets to verify if the original models could be correctly identified, using random-effects Bayesian model selection. We averaged the resulting PEPs across the 20 simulation seeds, creating a model confusion matrix (Figure S2H).

Our model fitting used the same priors from previous work (Table S11) ([Hauke et al., 2024](#SupHauke2024); [Karvelis et al., 2024](#SupKarvelis2024)). Random-effects Bayesian selection confirmed model 2a, the mean-reverting HGF with free parameters, as the winning model (Figure S1)**.** The average model recovery over the 20 simulations was 0.69 ± 0.37 for the standard HGF (model 1a), 0.21 ± 0.20 for the control standard HGF (model 1b), 0.99 ± 0.02 for the mean-reverting HGF (model 2a), and 0.98 ± 0.05 for the control mean-reverting HGF (model 2b). The poor recovery of control model 1b likely arises because the Bayes-optimal equilibrium parameter (*m*_3_) is close to the prior mean (µ3), causing the predictions of the Bayes-optimal standard and mean-reverting models to be highly similar. The drift component in the mean-reverting model exerts a stronger influence on behavioral predictions when the equilibrium parameter (*m*_3_) substantially deviates (either higher or lower) from the prior mean ([Hauke et al., 2024](#SupHauke2024)). Model recovery for a single simulation is depicted in Figure S2H.

## *Parameter Recovery*

We conducted a parameter recovery analysis to obtain an upper bound on the reliability of model parameter estimates ([Karvelis et al., 2023](#SupKarvelis2023)). This involved comparing the ‘simulated’ parameters (those used to generate the data) with the ‘recovered’ parameters (those estimated upon re-inverting the model on the same data). We assessed the accuracy of this recovery through Pearson correlations, p-values, and Cohen’s *f* ^2^, with an *f* ^2^ *≥* 0.35 indicating a large effect size ([Cohen, 2013](#Cohen2013)), which we interpreted as evidence for good parameter recovery in line with previous work ([Hauke et al., 2024](#SupHauke2024); [Karvelis et al., 2024](#SupKarvelis2024)).

Our parameter recovery analyses revealed good recovery for four out of seven parameters, with Cohen’s *f*^2^ *≥* 0.35 achieved in 100% of the simulations, notably including the drift equilibrium point *m*_3_ (mean *r* = 0.842 ± 0.028; Figure S2). Recovery for $\mu_{2}^{\left( 0 \right)}$, $\mu_{3}^{\left( 0 \right)}$, and $\kappa_{2}$ met the criterion for 95% (mean *r* = 0.649 ± 0.075), 40% (mean *r* = 0.427 ± 0.138) and 70% (mean *r* = 0.573 ± 0.075) of the simulations, respectively. Correlations between model parameters are detailed in Figure S4, and average parameter estimates across participants are presented in Table S12. Almost all correlations were unconcerning (|r|<0.6; Hauke et al., 2024), except for the correlation between $\kappa_{2}$ and $\omega_{2}$ (r=0.77).

Interestingly, this contrasts previous studies in healthy controls where the standard HGF without drift was favored ([Hauke et al., 2024](#Hauke2024)). This difference may reflect broader variability in learning strategies across our larger participant sample, thus necessitating the drift parameter ([Fromm et al., 2023](#Fromm2023)).

*Comparison with Simpler Models*

To evaluate whether simpler models could adequately capture participants’ behaviour in the social learning task, we performed an additional analysis by expanding the model space to include two simpler candidates: a Rescorla–Wagner model and a non-hierarchical two-level HGF. The priors used for these models were identical to those reported in Hauke et al. (2024, Table S1). Consistent with earlier work (Cole et al., 2020; Diaconescu et al., 2014, 2017, 2020; Hauke et al., 2024), model comparison favoured the models presented in the main manuscript (Figure S6).

*Social Bias Parameter Analysis*

We examined the distribution of the social bias parameter ζ from the winning mean-reverting HGF model. ζ reflects the relative weighting of social (advice) versus non-social (cue) information, with values closer to 1 indicating a stronger bias toward advice. Across participants, the mean ζ was 0.428 (SD = 0.150) (Figure S7). A one-sample t-test against 0.5 revealed a significant bias toward non-social cues, t_(42)_ = –3.14, p = 0.0031. This indicates that, on average, participants relied more heavily on individual non-social cues than on advice.

*Cannabis Consumption Analysis*

The sample included elevated rates of cannabis use, as healthy controls were recruited to match patients with early psychosis, who typically report higher cannabis consumption. To assess potential effects of cannabis on model-based and outcome-related EEG measures, cannabis use was included as a covariate in separate general linear models for each computational variable. A significant effect was observed for volatility PE (δ₂) at 434 ms post-stimulus (F₄₁ = 34.35, p = 0.005). No other significant effects emerged for computational trajectories or outcome-related EEG responses.

# Supplementary Figures
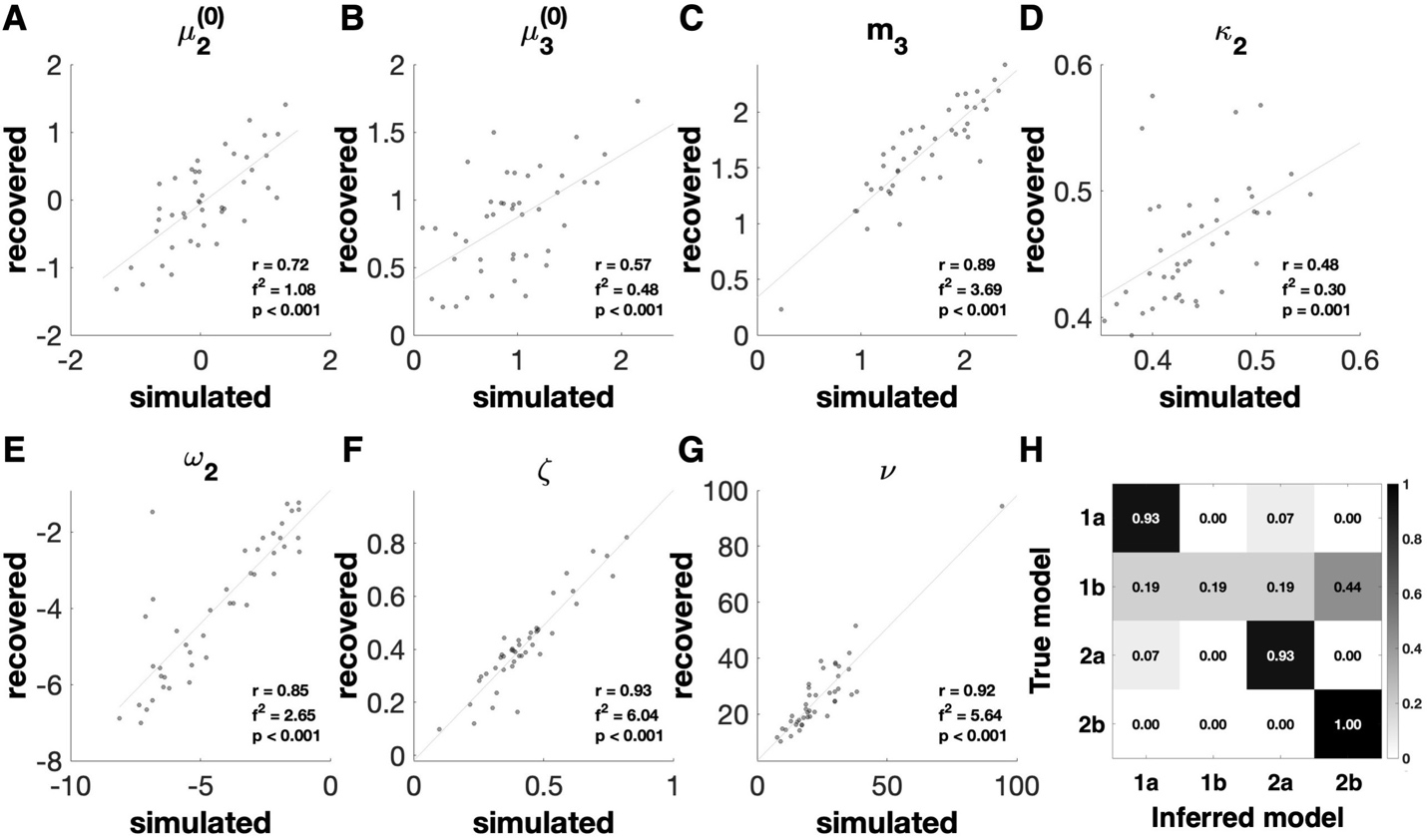


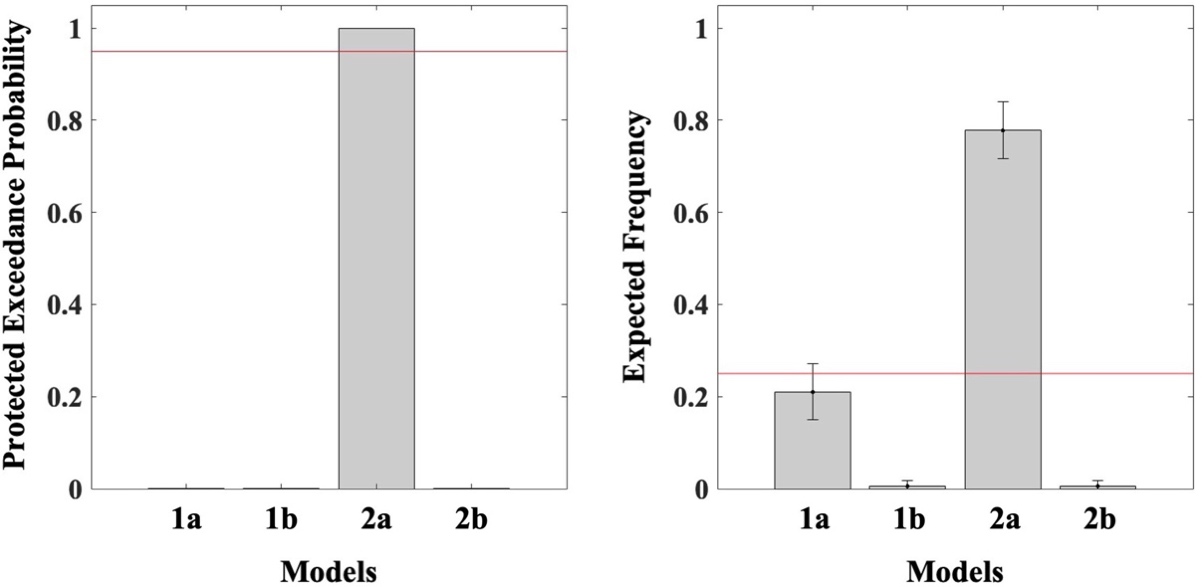


Figure S1: **Random-effects Bayesian Model Selection**. Standard HGF with free parameters (Model 1a) or Bayes optimal parameters (Model 1b) and mean-reverting HGF with free parameters (Model 2a) or Bayes optimal parameters (Model 2b).

Figure S2: **Parameter Recovery. A-G:** Results of parameter recovery for a single random seed in the mean-reverting Hierarchical Gaussian Filter (HGF) with drift at the 3rd level. H: The confusion matrix represents the average protected exceedance probability, computed across 20 datasets. Each dataset was simulated using identical parameters but varied by random seeds to incorporate stochastic variation.


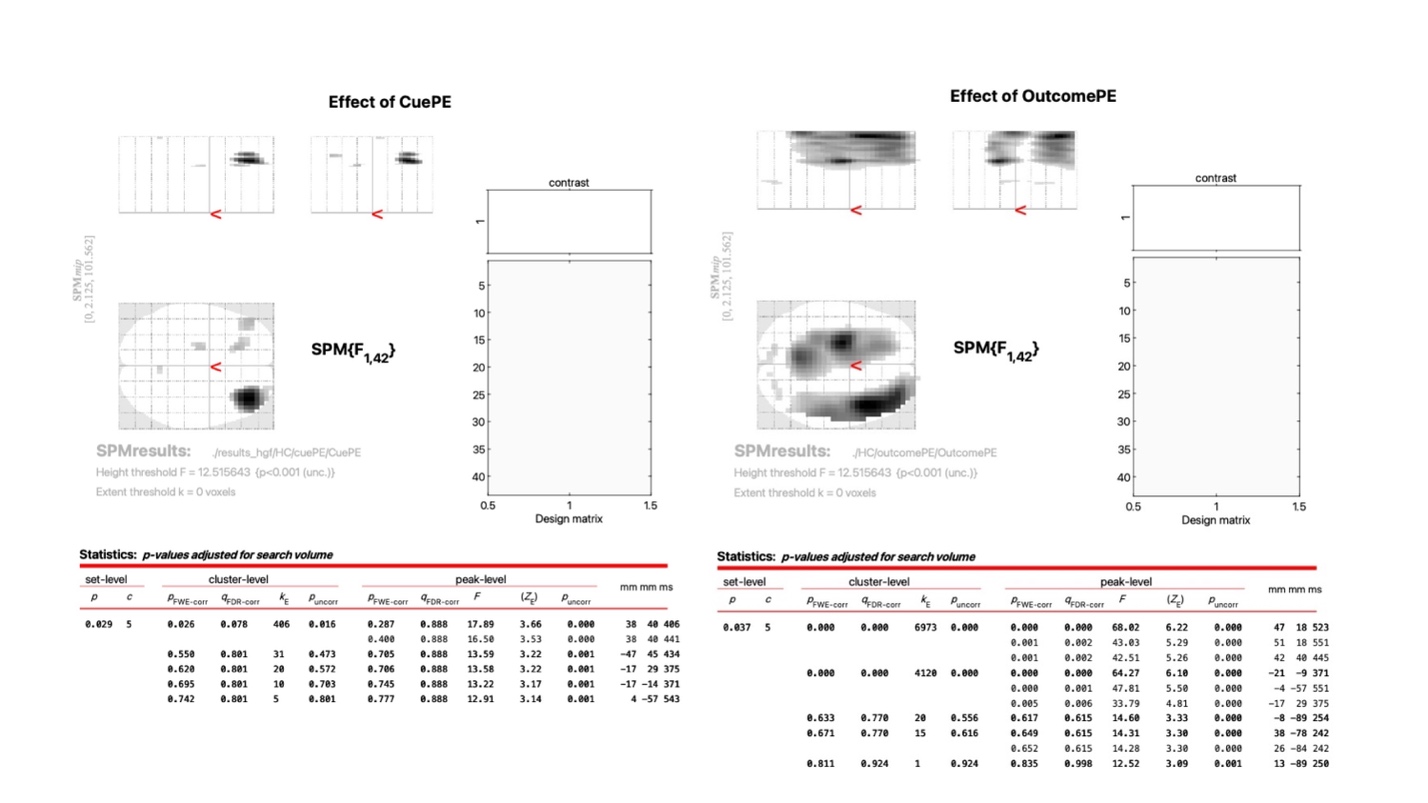


Figure S3: **Sensor-level effects of cue PE and outcome PE when modeled in separate GLMs.**


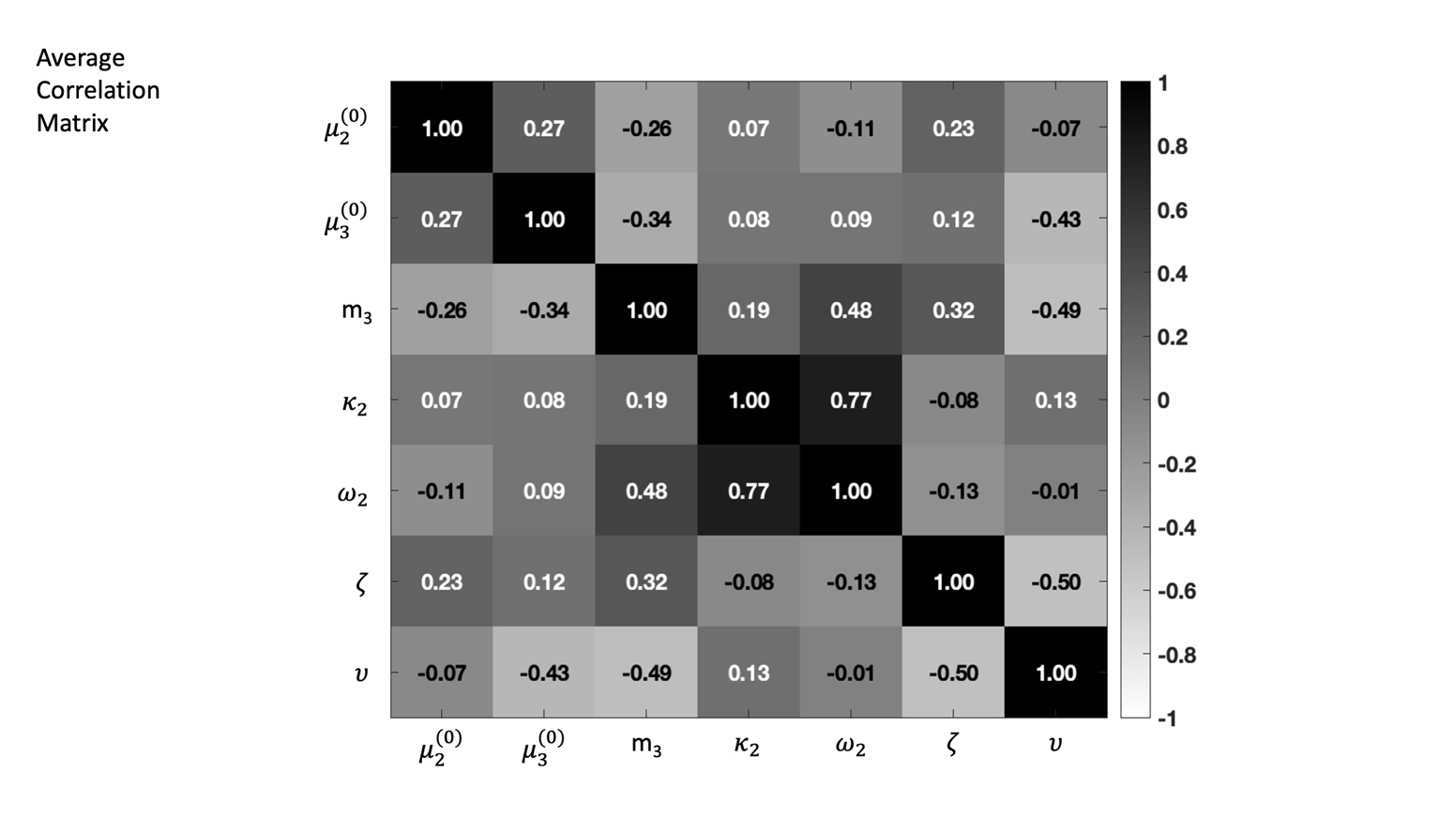


Figure S4: **Parameter Correlation Matrix.**


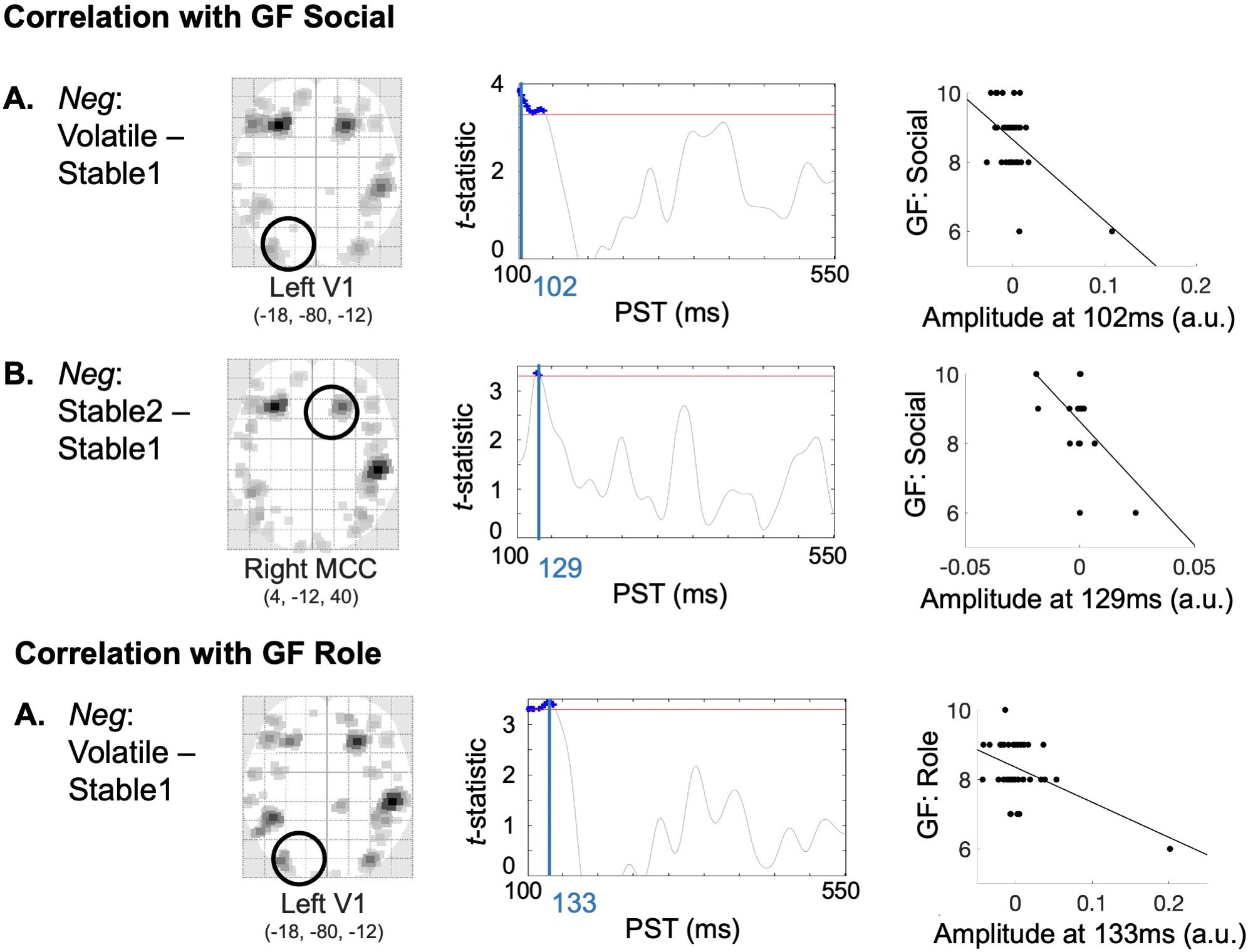


Figure S5: **Cortical sources for outcome-related response and functioning. A:** A negative correlation between the grand- averaged phase difference waveform (volatile phase - initial stable phase) and social function, with a maximal peak at 102ms in the left primary visual cortex (V1). Source activations are depicted on an SPM-glass brain in neurological orientation (left). Significant t-contrasts between the outcome difference waveform and global function (GF) scores over peri-stimulus time (PST) are displayed in the middle. A red horizontal line indicates the significance threshold under peak-family wise error (FWE) correction, with all surpassing time points in blue. The peak time point is highlighted by a blue vertical line. The scatter plot (right) displays the correlation between GF scores and the amplitude of the outcome difference waveform at the peak time point. B: Source activation of grand-averaged phase difference waveform (second stable phase - first stable phase) and social function, peaking at 129ms in the right middle cingulate cortex (MCC). C: Source activation of grand-averaged phase difference waveform (volatile phase - initial stable phase) and role function, peaking at 133ms in the left V1. Note: After removing the outlier participant with low GF scores and large amplitude values, the previously observed correlations were no longer statistically significant.


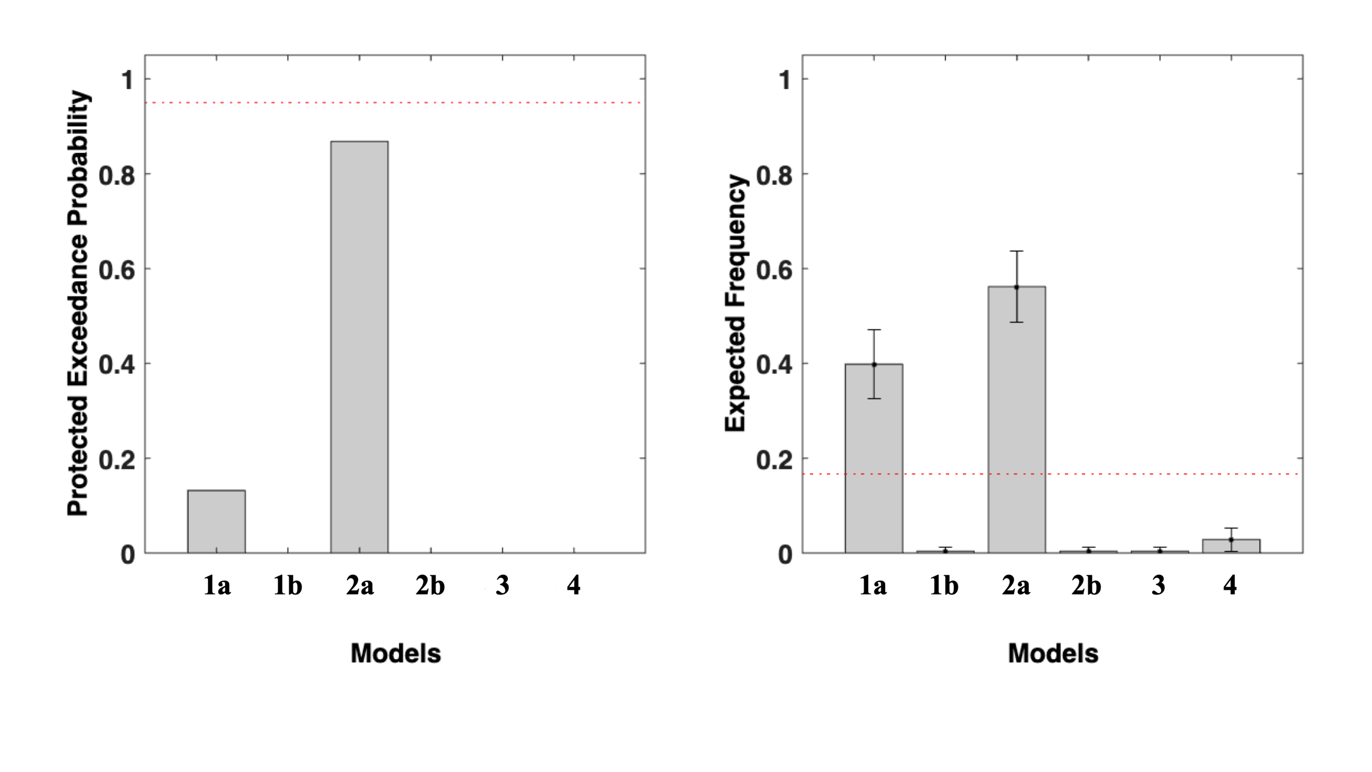


Figure S6**: Random-effects Bayesian Model Selection with Simpler Models.** Standard HGF with free parameters (Model 1a) or Bayes optimal parameters (Model 1b) and mean-reverting HGF with free parameters (Model 2a) or Bayes optimal parameters (Model 2b), a 2-level HGF (Model 3) and a Rescorla-Wagner model (Model 4) were included in the simpler model comparison.


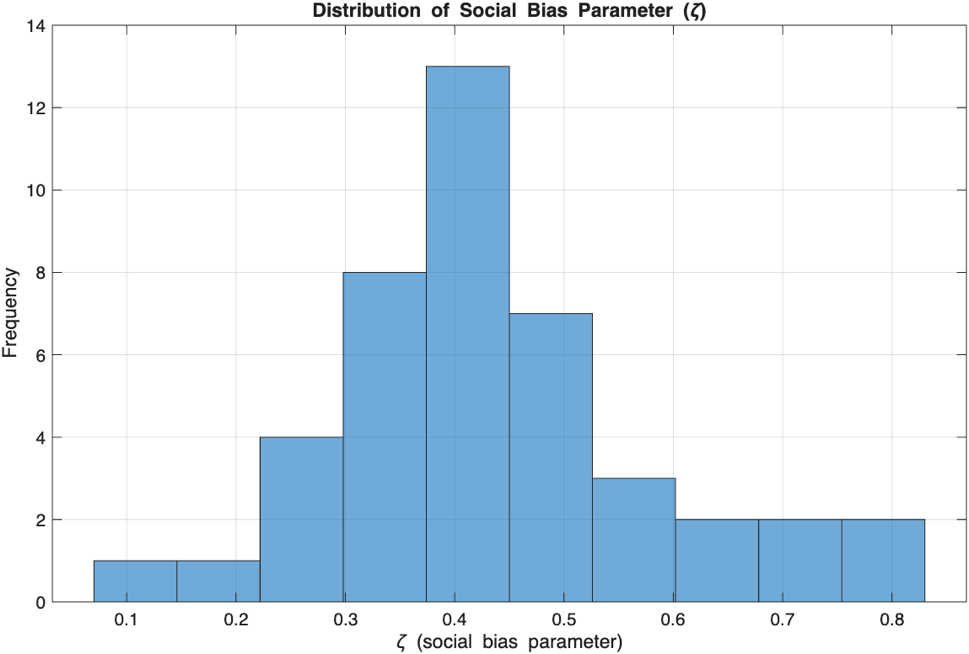


Figure S7:  **Distribution of the social bias parameter (ζ) across participants**

# Supplementary Tables

Table S1: Montreal Neurological Institute (MNI) coordinates of predefined sources.

| Source | Hemisphere | x | y | z |
| --- | --- | --- | --- | --- |
| Anterior temporoparietal junction | L | -52 | -50 | 30 |
|  | R | 52 | -50 | 30 |
| Cuneus | L | -12 | -96 | -2 |
|  | R | 14 | -90 | -2 |
| Dorsal lateral prefrontal cortex | L | -48 | 8 | 24 |
|  | R | 46 | 22 | 42 |
| Dorsal anterior cingulate cortex | L | -6 | 29 | 54 |
|  | R | 6 | 29 | 54 |
| Fusiform gyrus | L | -38 | -50 | -18 |
|  | R | 38 | -50 | -18 |
| Inferior occipital gyrus | L | -48 | -76 | -4 |
|  | R | -46 | -68 | -4 |
| Insula | L | -38 | 18 | -5 |
|  | R | 38 | 18 | -5 |
| Intraparietal sulcus | L | -30 | -50 | 46 |
|  | R | 30 | -50 | 46 |
| Inferior temporal gyrus | R | 58 | -30 | -28 |
| Inferior parietal lobule | L | -24 | -68 | 40 |
|  | R | 26 | -62 | 38 |
| Lingual Gyrus | L | -12 | -68 | 8 |
|  | R | 6 | -70 | 10 |
| Midcingulate Cortex | L | 0 | -10 | 28 |
|  | R | 4 | -12 | 40 |
| Posterior cingulate cortex | L | -8 | -46 | 28 |
|  | R | 8 | -46 | 28 |
| Rostral anterior cingulate cortex | L | -2 | 45 | 3 |
|  | R | 2 | 46 | 4 |
| Superior frontal gyrus | R | 4 | -22 | 54 |
| Supplementary motor area | L | -4 | 12 | 54 |
|  | R | 6 | 8 | 58 |
| Supramarginal gyrus | L | -48 | -32 | 54 |
|  | R | 62 | -20 | 26 |
| Superior parietal lobule | R | 38 | -42 | 42 |
| Temporoparietal junction | L | -34 | -46 | 42 |
|  | R | 30 | -66 | 42 |
| Primary visual cortex | L | -18 | -80 | -12 |
|  | R | 18 | -80 | -12 |

Table S2: Sensor-level F-test statistics for phase effects.

| Phase response | Cluster size (voxels) | Cluster p-value (FWE-corrected) | Peak p-value (FWE-corrected) | *F*84  (peak value) | Peak location (mm, mm, ms) |
| --- | --- | --- | --- | --- | --- |
| Stable 1 vs. Volatile | 631 | 0.009 | 0.010 | 25.81 | 8, -73, 297 |
|  | 409 | 0.033 | 0.133 | 18.15 | -4, 18, 328 |
| Stable 1 vs. Stable 2 | 617 | 0.010 | 0.018 | 23.99 | 4, -73, 293 |
|  | 460 | 0.024 | 0.094 | 19.18 | -4, 24, 250 |

Table S3: Sensor-level t-test statistics for phase effects.

| Phase response | Cluster size (voxels) | Cluster p-value (FWE-corrected) | Peak p-value (FWE-corrected) | *t*84  (peak value) | Peak location (mm, mm, ms) |
| --- | --- | --- | --- | --- | --- |
| Stable 1 > Volatile | 1069 | 0.002 | 0.005 | 5.08 | 8, -73, 297 |
| Stable 1 < Volatile | 645 | 0.014 | 0.069 | 4.26 | -4, 18, 328 |
| Stable 1 >  Stable 2 | 848 | 0.006 | 0.009 | 4.90 | 4, -73, 293 |
| Stable 1 < Stable 2 | 1023 | 0.003 | 0.048 | 4.38 | -4, 24, 250 |
|  | 465 | 0.034 | 0.052 | 4.35 | -26, -14, 398 |

Table S4: Summary of source-level t-statistics for phase difference waveform.

| Source Cluster size Cluster p-value  (voxels) (FWE-corrected) | Peak p-value (FWE-corrected) | *t*_84_  (peak value) | Peak time  (ms) |
| --- | --- | --- | --- |
| **Stable 1 > Volatile** |  |  |  |
| Right V1 1 0.018 | 0.017 | 3.22 | 266 |
| **Stable 1 < Stable 2** |  |  |  |
| Left FG 4 0.014 | 0.004 | 3.73 | 215 |
| Left IPL 7 0.008 | 0.008 | 3.42 | 258 |
| **Stable 1 > Stable 2** |  |  |  |
| Left PCC 7 0.007 | 0.006 | 3.62 | 145 |
| Right PCC 7 0.007 | 0.006 | 3.60 | 141 |

Table S5: Summary of sensor-level statistics for peak-level effects (p < 0.05, whole-volume FWE-corrected) of activation for computational trajectories. *Note, cluster-level effects (p < 0.001, uncorrected) reported for volatility precision.

| Computational | Cluster size | Cluster p-value | Peak p-value | *F*42 | Cohen’s | Peak location | |
| --- | --- | --- | --- | --- | --- | --- | --- |
| parameter | (voxels) | (FWE-corrected) | (FWE-corrected) | (peak value) | F2 | (mm, mm, ms) | |
| Cue PE (*δ_c_*) | 499 | 0.015 | 0.015 | 29.30 |  | | 13, -25, 441 |
| Outcome | 3070 | *<*0.001 | *<*0.001 | 44.68 |  | | 0, -46, 535 |
| PE (*δ_b_*) |  |  |  |  |  | |  |
| Absolute  Advice | 4898 | *<*0.001 | 0.001 | 41.49 |  | | 8, -30, 445 |
| PE (*δ*_1_) | 4127 | *<*0.001 | 0.004 | 35.50 |  | | 55, -41, 441 |
| Signed  Advice | 1138 | *<*0.001 | 0.003 | 36.56 |  | | 55, -46, 527 |
| PE (*δ*_1_) | 1613 | *<*0.001 | 0.017 | 29.12 |  | | -4, -46, 512 |
| Volatility | 2712 | *<*0.001 | 0.003 | 36.42 |  | | 0, -41, 535 |
| PE (*δ*_2_) | 2028 | *<*0.001 | 0.042 | 25.64 |  | | 60, -3, 531 |
| *Volatility | 339 | 0.047 | 0.195 | 19.17 |  | | 0, 29, 328 |
| Precision (*π*_3_) |  |  |  |  |  | |  |

Table S6: Summary of source-level statistics of significant clusters of activation for computational trajectories.

| Source | Cluster size | Cluster p-value | Peak p-value | *t*42 | Cohen’s | Peak time | |
| --- | --- | --- | --- | --- | --- | --- | --- |
| parameter | (voxels) | (FWE-corrected) | (FWE-corrected) | (peak value) | F2 | (ms) | |
| **Absolute Advice PE (*δ*_1_)** | | | | | | | |
| Right Insula  (pos. effect) | 4 | 0.014 | 0.014 | 3.47 |  | | 391 |
| **Volatility PE (*δ*_2_)** | | | | | | | |
| Left MCC  (neg. effect) | 2 | 0.022 | 0.023 | 3.34 |  | | 266 |
| **Volatility Precision (**$\boldsymbol{\pi}_{\boldsymbol{3}}$**)** | | | | | | | |
| Right LG  (neg. effect) | 3 | 0.016 | 0.014 | 3.42 |  | | 137 |

Table S7: Summary of source-level statistics for associations between global function and phase difference waveform.

| Source Cluster size Cluster p-value  (voxels) (FWE-corrected) | | | Peak p-value (FWE-corrected) | *t*_41_  (peak value) | Peak time  (ms) |
| --- | --- | --- | --- | --- | --- |
| **Social Function: Volatile – Stable 1** | | |  |  |  |
| Left V1*  (neg. effect) 10 0.003 | | | 0.005 | 3.84 | 102 |
| Left FG 6 0.009 | | | 0.004 | 3.95 | 398 |
| (pos. effect) 8 0.004 | | | 0.010 | 3.61 | 121 |
| 3 0.017 | | | 0.016 | 3.41 | 520 |
| **Social Function: Stable 2 – Stable 1** | | |  |  |  |
| Right MCC*  (neg. effect) | 2 | 0.022 | 0.021 | 3.36 | 129 |
| **Social Function: Stable 2 – Volatile** | | | | | |
| Left FG  (neg. effect) | 5 | 0.012 | 0.002 | 4.12 | 102 |
| Right CC*  (neg. effect) | 7 | 0.008 | 0.007 | 3.64 | 543 |
| Right V1*  (pos. effect) | 4 | 0.014 | 0.008 | 3.63 | 121 |
| Right IPL  (pos. effect) | 3 | 0.016 | 0.008 | 3.64 | 340 |
| **Role Function: Volatile – Stable 1** | | |  |  |  |
| Left V1*  (neg. effect) | 10 | 0.003 | 0.013 | 3.45 | 133 |
| Left FG 18 *<*0.001 | | | 0.001 | 4.40 | 398 |
| (pos. effect) 10 0.002 | | | 0.002 | 4.27 | 516 |
| **Role Function: Stable 2 – Stable 1** | | |  |  |  |
| Left IPL  (pos. effect) | 6 | 0.010 | 0.011 | 3.47 | 258 |
| **Role Function: Stable 2 – Volatile** | | | | | |
| Left DLPFC  (neg. effect) | 25  8 | *<*0.001  0.006 | 0.002  0.011 | 3.68  3.23 | 156  234 |
| Left FG  (neg. effect) | 12 | *<*0.001 | 4.41 | 3.96 | 102 |
| Left IPS  (pos. effect) | 9  1 | 0.005  0.016 | 0.012  0.015 | 3.42  3.32 | 406  254 |
| Left FG*  (pos. effect) | 9 | 0.003 | 0.005 | 3.87 | 547 |

**Note: effects were driven by outlier subject.*

Table S8: Summary of sensor-level statistics for associations between global function and computational trajectories.

| Computational parameter | Cluster size  (voxels) | Cluster p-value (FWE-corrected) | Peak p-value (FWE-corrected) | *t*_41_  (peak value) | Peak time  (mm, mm, ms) |
| --- | --- | --- | --- | --- | --- |
| **Social Function** | | | | | |
| Advice Precision ($\pi_{2}$)  (neg. effect) | 123 | 0.242 | 0.009 | 5.38 | -42, -68, 129 |
| **Role Function** | | | | | |
| Advice Precision ($\pi_{2}$)  (neg. effect) | 138 | 0.216 | 0.041 | 4.79 | -8, 45, 449 |

Table S9: Summary of source-level statistics for associations between global role functioning and computational trajectories.

| Source | Cluster size  (voxels) | Cluster p-value  (FWE-corrected) | Peak p-value  (FWE-corrected) | *t*41  (peak value) | Peak time  (ms) |
| --- | --- | --- | --- | --- | --- |
| **Cue PE (**$\boldsymbol{\delta}_{\boldsymbol{c}}$**)** | | | | | |
| Left SMG  (neg. effect) | 4 | 0.014 | 0.015 | 3.46 | 230 |
| Left IOG  (neg. effect) | 11 | 0.001 | 0.011 | 3.56 | 500 |
| Left PCC  (neg. effect) | 1 | 0.020 | 0.021 | 3.30 | 305 |
| Right PCC | 9 | 0.003 | 0.002 | 4.13 | 387 |
| (neg. effect) | 15 | *<*0.001 | 0.008 | 3.78 | 309 |
|  | 13 | <0.001 | 0.010 | 3.56 | 500 |
| Left FG | 41 | *<*0.001 | 0.001 | 4.37 | 469 |
| (pos. effect) | 3 | 0.015 | 0.011 | 3.49 | 297 |
|  | 3 | 0.015 | 0.012 | 3.46 | 367 |
| **Outcome PE (**$\boldsymbol{\delta}_{\boldsymbol{c}}$**)** | | | | | |
| Left MCC  (neg. effect) | 4 | 0.014 | 0.012 | 3.31 | 289 |
| Left FG | 8 | 0.007 | 0.002 | 4.10 | 355 |
| (neg. effect) | 37 | *<*0.001 | 0.003 | 3.89 | 473 |
|  | 5 | 0.011 | 0.004 | 3.53 | 301 |
| Right IOG  (pos. effect) | 11 | 0.002 | 0.012 | 3.49 | 523 |
| Left PCC | 17 | *<*0.001 | 0.002 | 4.26 | 406 |
| (pos. effect) | 19 | *<*0.001 | 0.006 | 3.49 | 496 |
|  | 8 | 0.005 | 0.007 | 3.42 | 301 |
|  | 2 | 0.019 | 0.018 | 3.13 | 551 |
| Right PCC | 49 | *<*0.001 | *<*0.001 | 4.34 | 398 |
| (pos. effect) | 13 | 0.001 | 0.002 | 3.73 | 301 |
|  | 1 | 0.019 | 0.019 | 3.10 | 254 |
| **Advice PE (**$\boldsymbol{\delta}_{\boldsymbol{1}}$**)** | | | | | |
| Left FG | 38 | *<*0.001 | 0.002 | 3.94 | 430 |
| (neg. effect) | 6 | 0.009 | 0.003 | 3.87 | 297 |
|  | 4 | 0.011 | 0.008 | 3.51 | 352 |
|  | 1 | 0.012 | 0.010 | 3.48 | 102 |
| Right IOG  (pos. effect) | 16 | *<*0.001 | 0.005 | 3.77 | 520 |
| Left PCC | 18 | *<*0.001 | 0.002 | 4.18 | 379 |
| (pos. effect) | 4 | 0.013 | 0.014 | 3.39 | 293 |
| Right PCC | 18 | *<*0.001 | 0.001 | 4.30 | 379 |
| (pos. effect) | 6 | 0.010 | 0.009 | 3.57 | 293 |
|  | 22 | *<*0.001 | 0.010 | 3.52 | 508 |
| **Advice Precision (**$\boldsymbol{\pi}_{\boldsymbol{2}}$**)** | | | | | |
| Left V1 | 6 | 0.009 | 0.006 | 3.47 | 152 |
| (neg. effect) | 2 | 0.020 | 0.011 | 3.30 | 102 |
| Right V1 | 7 | 0.005 | 0.011 | 3.33 | 145 |
| (neg. effect) |  |  |  |  |  |
| Left LG | 9 | 0.003 | 0.005 | 3.82 | 336 |
| (neg. effect) | 12 | 0.001 | 0.012 | 3.51 | 520 |
|  | 6 | 0.009 | 0.013 | 3.49 | 148 |
| Left IOG | 24 | *<*0.001 | 0.001 | 3.98 | 250 |
| (neg. effect) | 41 | *<*0.001 | 0.001 | 3.93 | 414 |
| Right IOG | 6 | 0.010 | 0.005 | 3.70 | 348 |
| (neg. effect) | 7 | 0.009 | 0.007 | 3.56 | 410 |
| Right PCC  (neg. effect) | 1 | 0.020 | 0.020 | 3.32 | 141 |
| Left FG | 9 | 0.003 | 0.001 | 4.52 | 406 |
| (pos. effect) | 7 | 0.007 | 0.003 | 4.04 | 348 |
|  | 18 | *<*0.001 | 0.003 | 3.98 | 547 |
|  | 13 | *<*0.001 | 0.008 | 3.65 | 102 |
| **Volatility PE (**$\boldsymbol{\delta}_{\boldsymbol{2}}$**)** | | | | | |
| Left FG | 42 | <0.001 | 0.001 | 4.21 | 430 |
| (neg. effect) | 9 | 0.007 | 0.001 | 4.16 | 352 |
|  | 10 | 0.006 | 0.002 | 3.90 | 297 |
|  | 4 | 0.010 | 0.003 | 3.80 | 102 |
| Left V1  (pos. effect) | 4  1 | 0.014  0.018 | 0.015  0.018 | 3.17  3.10 | 168  309 |
| Left IOG | 12 | 0.001 | <0.001 | 4.99 | 422 |
| (pos. effect) | 3 | 0.017 | 0.008 | 3.39 | 355 |
| Right IOG | 17 | <0.001 | <0.001 | 4.52 | 422 |
| (pos. effect) | 18 | <0.001 | 0.006 | 3.43 | 520 |
|  | 3 | 0.015 | 0.015 | 3.15 | 352 |
| Left PCC | 4 | 0.013 | 0.011 | 3.46 | 371 |
| (pos. effect) | 11 | 0.003 | 0.011 | 3.46 | 418 |
| **Volatility Precision (**$\boldsymbol{\pi}_{\mathbf{3}}$**)** | | | | | |
| Left FG | 8 | 0.005 | <0.001 | 4.71 | 398 |
| (neg. effect) | 3 | 0.017 | 0.011 | 3.54 | 102 |
|  | 8 | 0.005 | 0.014 | 3.45 | 543 |
|  | 3 | 0.017 | 0.019 | 3.34 | 344 |
| Right V1  (pos. effect) | 2 | 0.019 | 0.020 | 3.32 | 148 |
| Left IOG  (pos. effect) | 2 | 0.017 | 0.014 | 3.39 | 312 |
| Left CC  (pos. effect) | 2 | 0.014 | 0.010 | 3.47 | 102 |
| Right CC | 7 | 0.006 | 0.008 | 3.69 | 152 |
| (pos. effect) | 4 | 0.014 | 0.011 | 3.59 | 332 |
| Left LG | 9 | 0.004 | 0.005 | 3.82 | 398 |
| (pos. effect) | 5 | 0.012 | 0.008 | 3.64 | 340 |
|  | 14 | <0.001 | 0.009 | 3.62 | 539 |
| Left PCC  (pos. effect) | 3 | 0.017 | 0.018 | 3.36 | 121 |
| Right PCC | 6 | 0.009 | 0.013 | 3.48 | 117 |
| 7(pos. effect) | 8 | 0.004 | 0.014 | 3.46 | 156 |

Table S10: Summary of source-level summary statistics for associations between global social functioning and computational trajectories.

| Source | Cluster size  (voxels) | Cluster p-value  (FWE-corrected) | | | Peak p-value  (FWE-corrected) | | *t*41  (peak value) | | Peak time  (ms) |
| --- | --- | --- | --- | --- | --- | --- | --- | --- | --- |
| **Cue PE (**$\boldsymbol{\delta}_{\boldsymbol{c}}$**)** | | | | | | | | | |
| Left CC  (pos. effect) | 3 | 0.016 | | | 0.005 | | 3.79 | | 246 |
| **Outcome PE (**$\boldsymbol{\delta}_{\boldsymbol{c}}$**)** | | | | | | | | | |
| Right V1  (pos. effect) | 29 | <0.001 | | | 0.003 | | 4.02 | | 469 |
| Right CC  (pos. effect) | 3 | 0.017 | | | 0.010 | | 3.58 | | 227 |
| **Advice PE (**$\boldsymbol{\delta}_{\boldsymbol{1}}$**)** | | | | | | | | | |
| Left V1  (pos. effect) | 26 | <0.001 | | | 0.007 | | 3.66 | | 473 |
| Right V1  (pos. effect) | 32 | <0.001 | | | 0.003 | | 3.97 | | 465 |
| Right CC  (pos. effect) | 5 | 0.012 | | | 0.005 | | 3.79 | | 223 |
| Left LG  (pos. effect) | 9 | 0.004 | | | 0.012 | | 3.46 | | 410 |
| Right PCC  (pos. effect) | 1 | 0.017 | | | 0.017 | | 3.30 | | 207 |
| Left SMA  (pos. effect) | 2 | 0.018 | | | 0.018 | | 3.35 | | 234 |
| **Advice Precision (**$\boldsymbol{\pi}_{\boldsymbol{2}}$**)** | | | | | | | | | |
| Left CC  (neg. effect) | 7 | 0.007 | | | 0.012 | | 3.52 | | 133 |
| Left LG  (neg. effect) | 13 | 0.001 | | | 0.001 | | 3.94 | | 152 |
| Right LG  (neg. effect) | 6 | 0.008 | | | 0.010 | | 3.63 | | 148 |
| Left PCC | 12 | 0.001 | | | 0.005 | | 3.84 | | 125 |
| (neg. effect) |  |  | | |  | |  | |  |
| Right PCC | 13 | *<*0.001 | | | 0.003 | | 4.02 | | 129 |
| (neg. effect) |  |  | | |  | |  | |  |
| Left FG | 17 | *<*0.001 | | | 0.009 | | 3.61 | | 512 |
| (pos. effect) | 14 | <0.001 | | | 0.010 | | 3.58 | | 121 |
| **Volatility PE (**$\boldsymbol{\delta}_{\boldsymbol{2}}$**)** | | | | | | | | | |
| Left FG  (neg. effect) | 11 | 0.005 | | | 0.008 | | 3.42 | | 422 |
| Left V1  (pos. effect) | 24 | *<*0.001 | | | 0.006 | | 3.73 | | 531 |
| Right V1 | 4 | | 0.014 | 0.005 | | 3.78 | | 223 | |
| (pos. effect) | 7 | | 0.007 | 0.017 | | 3.35 | | 531 | |
|  | 2 | | 0.018 | 0.019 | | 3.30 | | 492 | |
| Right CC  (pos. effect) | 6 | 0.009 | | | 0.010 | | 3.55 | | 418 |
| Left LG | 11 | 0.002 | | | 0.007 | | 3.65 | | 422 |
| (pos. effect) | 1 | 0.018 | | | 0.010 | | 3.54 | | 102 |
|  | 4 | 0.014 | | | 0.011 | | 3.48 | | 316 |
|  | 4 | 0.014 | | | 0.012 | | 3.47 | | 172 |
|  | 8 | 0.006 | | | 0.015 | | 3.36 | | 492 |
| Right LG | 1 | 0.020 | | | 0.014 | | 3.43 | | 102 |
| (pos. effect) | 1 | 0.020 | | | 0.020 | | 3.31 | | 172 |
| Left IOG  (pos. effect) | 11 | 0.001 | | | 0.008 | | 3.66 | | 414 |
| Left PCC | 6 | 0.010 | | | 0.011 | | 3.47 | | 312 |
| (pos. effect) | 4 | 0.013 | | | 0.013 | | 3.39 | | 430 |
| Right PCC | 8 | 0.006 | | | 0.009 | | 3.56 | | 426 |
| (pos. effect) | 7 | 0.008 | | | 0.009 | | 3.54 | | 309 |
| **Volatility Precision (**$\boldsymbol{\pi}_{\mathbf{3}}$**)** | | | | | | | | | |
| Left V1  (pos. effect) | 4 | 0.014 | | | 0.012 | | 3.24 | | 125 |
| Right V1  (pos. effect) | 1 | 0.021 | | | 0.017 | | 3.39 | | 332 |
| Left LG  (pos. effect) | 2 | 0.017 | | | 0.018 | | 3.32 | | 148 |
| Right LG  (pos. effect) | 2 | 0.018 | | | 0.016 | | 3.37 | | 145 |
| Left PCC  (pos. effect) | 3 | 0.017 | | | 0.011 | | 3.56 | | 230 |
| Right PCC  (pos. effect) | 3 | 0.017 | | | 0.017 | | 3.37 | | 137 |

Table S11: Priors on free model parameters.

|  | Equilibrium  Point | Coupling  Strength | Evolution  Rate | Prior  Expectations | Advice  Weight | Decision  Noise |
| --- | --- | --- | --- | --- | --- | --- |
| Model 1 |  | $\kappa_{2}(logit(0.5, 1), 1)$ | $\omega_{2}(-2, 4)$ | $\mu_{2}^{(0)}(0, 1), \mu_{3}^{(0)}(1, 1)$ | $\zeta(logit(0.5, 1), 1)$ | $\nu(log(48), 1)$ |
| Model 2 | m3(1,1) | $\kappa_{2}(logit(0.5, 1), 1)$ | $\omega_{2}(-2, 4)$ | $\mu_{2}^{(0)}(0, 1), \mu_{3}^{(0)}(1, 1)$ | $\zeta(logit(0.5, 1), 1)$ | $\nu(log(48), 1)$ |

Table S12: Average Parameter Value Across Participants (n=43)

| **Parameter** | **Average Value** |
| --- | --- |
| $\mu_{2}^{(0)}$ | 0.0793 |
| $\mu_{3}^{(0)}$ | 0.9407 |
| m_3_ | 1.6124 |
| $\kappa_{2}$ | 0.4401 |
| $\omega_{2}$ | 4.3703 |
| $\zeta$ | 0.4284 |
| $\nu$ | 24.2404 |

# Supplementary References

1. Cohen J (2013) Statistical power analysis for the behavioral sciences, 2nd ed. Routledge. <https://doi.org/10.4324/9780203771587>
2. Cole DM, Diaconescu AO, Pfeiffer UJ, Brodersen KH, Mathys CD, Julkowski D, Ruhrmann S, Schilbach L, Tittgemeyer M, Vogeley K, et al. (2020) **Atypical processing of uncertainty in individuals at risk for psychosis** *NeuroImage: Clinical* **26**:102239. <https://doi.org/10.1016/j.nicl.2020.102239>
3. Diaconescu AO, Mathys C, Weber LA, Daunizeau J, Kasper L, Lomakina EI, Fehr E, Stephan KE (2014) **Inferring on the intentions of others by hierarchical Bayesian learning** *PLoS Computational Biology* **10**:e1003810. <https://doi.org/10.1371/journal.pcbi.1003810>
4. Diaconescu AO, Mathys C, Weber LA, Kasper L, Mauer J, Stephan KE (2017) **Hierarchical prediction errors in midbrain and septum during social learning** *Social Cognitive and Affective Neuroscience* **12**:618–34. <https://doi.org/10.1093/scan/nsw171>
5. Diaconescu AO, Wellstein KV, Kasper L, Mathys C, Stephan KE (2020) **Hierarchical Bayesian models of social inference for probing persecutory delusional ideation.** *Journal of abnormal psychology* **129**:556-569. https://doi.org/10.1037/abn0000500
6. Fromm S, Katthagen T, Deserno L, Heinz A, Kaminski J, Schlagenhauf F (2023) **Belief updating in subclinical and clinical delusions.** *Schizophrenia Bulletin Open* **4**:sgac074. <https://doi.org/10.1093/schizbullopen/sgac074>.
7. Hauke DJ, Roth V, Karvelis P, Adams RA, Moritz S, Borgwardt S, Diaconescu AO, Andreou C (2022) **Increased belief instability in psychotic disorders predicts treatment response to metacognitive training** *Schizophrenia Bulletin* **48**:826–38. <https://doi.org/10.1093/schbul/sbac029>
8. Hauke DJ, Wobmann M, Andreou C, Mackintosh AJ, de Bock R, Karvelis P, Adams RA, Sterzer P, Borgwardt S, Roth V, et al. (2024) **Altered perception of environmental volatility during social learning in emerging psychosis** *Computational Psychiatry* **8**:1. <https://doi.org/10.5334/cpsy.95>
9. Karvelis P, Hauke DJ, Wobmann M, Andreou C, Mackintosh A, de Bock R, Borgwardt S, Diaconescu AO (2024) **Test-retest reliability of behavioral and computational measures of advice taking under volatility.** *PLOS ONE* **19**:e0312255. <https://doi.org/10.1371/journal.pone.0312255>
10. Karvelis P, Paulus MP, Diaconescu AO (2023) **Individual differences in computational psychiatry: A review of current challenges.** *Neuroscience & Biobehavioral Reviews* **148**:105137. <https://doi.org/10.1016/j.neubiorev.2023.105137>
11. Lehrl S, Triebig G, Fischer BANS (1995) **Multiple choice vocabulary test MWT as a valid and short test to estimate premorbid intelligence.** *Acta Neurologica Scandinavica* **91**:335–345. <https://doi.org/10.1111/j.1600-0404.1995.tb07018.x>
